# Structure and Optical Properties of New 2-*N*-Phenylamino-methyl-nitro-pyridine Isomers

**DOI:** 10.3390/ijms26072874

**Published:** 2025-03-21

**Authors:** Patrycja Godlewska, Jerzy Hanuza, Jan Janczak, Radosław Lisiecki, Małgorzata Basiak, Adam Zając, Lucyna Dymińska

**Affiliations:** 1 Department of Bioorganic Chemistry, Faculty of Production Engineering, Wroclaw University of Economics and Business, 118-120 Komandorska Str., 53-345 Wrocław, Poland; 184180@student.ue.wroc.pl (M.B.); adam.zajac@ue.wroc.pl (A.Z.); 2 Institute of Low Temperature and Structure Research, 2 Okólna Str., 50-422 Wrocław, Poland; j.janczak@intibs.pl (J.J.); r.lisiecki@intibs.pl (R.L.);

**Keywords:** 2-*N*-[phenylamino-methyl-nitropyridines], crystal structure, IR and Raman studies, electron absorption and luminescence spectra, DFT calculations, substitution effect of the methyl groups

## Abstract

Two new 2-*N*-phenylamino-(4 or 6)-methyl-3-nitropyridine derivatives were synthesized. Their structures were characterized on the basis of X-ray diffraction, IR, and Raman spectra as well as electron UV-Vis and emission spectra measurements. The experimental results were analyzed in terms of theoretical data in which the quantum chemical DFT and NBO calculations were applied. To elucidate the relaxation pathways of electronically excited states, multiple excitation wavelengths were employed to probe energy dissipation mechanisms in the studied compounds. A systematic analysis was conducted to evaluate how variations in methyl substituent positioning modulate both the structural architecture and photophysical behavior of the isomeric systems. The spectroscopic, structural and theoretical considerations allow us to propose the potential technological applications derived from the unique properties of these isomers.

## 1. Introduction

The synthesis and investigation of new 2-*N*-phenylamino-methyl-nitropyridines have garnered significant attention due to their versatile properties and potential applications across various industries. These compounds, characterized by their unique structural features and tunable chemical properties, have been explored extensively in the fields of material science, optoelectronics, and biochemistry. Additionally, they belong to a wide class of pyridine derivatives, which serve as a fundamental unit in many natural products, such as vitamins, alkaloids, coenzymes, drugs, and pesticides. Pyridine derivatives also exhibit a broad spectrum of biological activities, including anti-inflammatory [1,2], antiviral [3,4], anticancer [5,6,7,8,9], antimicrobial [10,11], antidiabetic [12,13], osteogenic [14,15], and antihypertensive [16] properties. Recent findings even highlight their potential antiviral activity against SARS-CoV-2 (COVID-19) and antifungal effects [17,18,19,20,21,22,23,24,25]. Due to their specific chemical properties, they were used as oxidizing reagents in organic syntheses [26,27] as well as the ligands for complexation of the metal ions [28,29].

Recent advancements have highlighted the potential use of dyes derived from pyridine compounds in the development of smart materials, particularly in packaging films. Laboratory experiments demonstrated that new derivatives of 2-*N*-phenylamino-3-nitro-6-methylpyridine exhibit promising properties when incorporated into chitosan-based polymers. These derivatives show excellent color intensity and stability, as well as low toxicity. Experimental trials produced chitosan films infused with these derivatives, showcasing not only their ease of integration into polymer matrices but also their ability to enhance the functional attributes of the final materials. The dyes dissolved well in chitosan gel, enabling efficient and economical coloring, which adds significant value for industrial applications.

In this study, we focus on the synthesis, structural characterization, and spectroscopic analysis of new pyridine derivatives, particularly 2-*N*-phenylamino-(4 or 6)-methyl-(3)-nitropyridine. The structural nuances of these compounds, elucidated through X-ray crystallography and supported by theoretical modeling, reveal insights into their bonding interactions and potential reactivity. Furthermore, the optical and electronic properties, explored through vibrational spectroscopy and UV-Vis analysis, underscore their versatility and suitability for incorporation into functional materials. The influence of methyl group substitution in the pyridine ring on the structure, electron level distribution, and spectroscopic properties was studied experimentally and theoretically using DFT and NBO quantum chemical calculations. These studies are a continuation of our works on a broad class of pyridine derivatives [30,31,32].

This research is a continuation of previous studies on pyridine derivatives [31,32], which have extensively characterized the vibrations and reactivity of compounds closely related to those investigated in this work. By aligning the findings of this research with practical applications, such as the incorporation of pyridine-derived dyes into chitosan films, this work bridges the gap between fundamental chemistry and applied science.

## 2. Results and Discussion

### 2.1. Structural Characterization

The effect of substituents in the pyridine ring of nitro and methyl groups in 2-*N*-phenylaminopyridine strongly affects the structural and optical properties of these derivatives. The red color is characteristic of isomers with a nitro group substituted in the 3-position of the pyridine ring. The position of substituents in these isomers is also reflected in their crystal structures.

#### 2.1.1. Description of the Structure of 2-*N*-Phenylamino-3-nitro-4-methylpyridine

**Figure 1:**
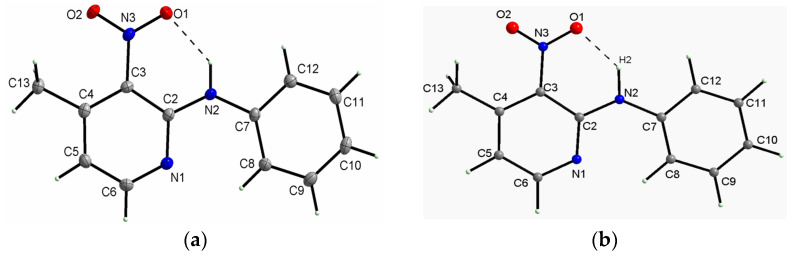
View of the X-ray structure of PA3N4MP (**a**) and DFT optimized molecule (**b**).

2-*N*-phenylamino-3-nitro-4-methylpyridine (PA3N4MP) crystallizes in the centrosymmetric space group of the triclinic system with two molecules per unit cell. Its X-ray molecular structure together with the optimized one, for comparison, is shown in Figure 1—selected geometrical parameters are listed in Table 1. The conformation of the 2-*N*-phenylamino-3-nitro-4-methylpyridine molecule is not planar. The planar NO_2_ group is slightly twisted in relation to the plane of the pyridine ring by 3.84(15)°, whereas the dihedral angle between the average plane of pyridine ring and the average plane of phenyl ring is 6.20(15)°. A weak intramolecular N–H∙∙∙O is observed. This is reflected in the N–O bond lengths of the NO_2_ group (Table 1).

The geometry of the 2-*N*-phenylamino-3-nitro-4-methylpyridine molecule in the gaseous phase obtained from the DFT calculations also shows a non-planar conformation. The dihedral angle between the plane of NO_2_ group and the plane of the pyridine ring of ~25° is significantly greater than that in the crystal. A quite similar correlation also occurs for the dihedral angle between the planes of the pyridine and phenyl rings between the conformations of the molecule in the gas phase and the solid phase. Selected geometrical DFT parameters are included in Table 1, but full details are listed in Table S1 (in the Supplementary Materials).

**Figure 2:**
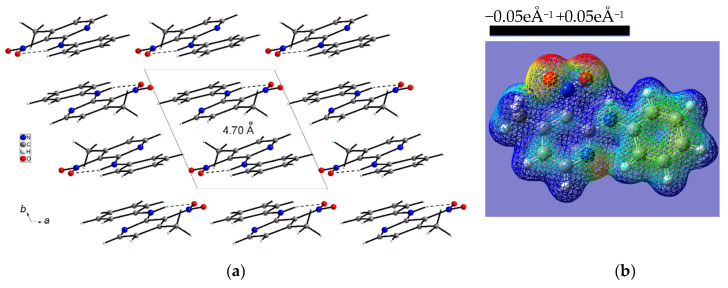
Arrangement of molecules in the unit cell showing the stacking structure along b-axis (**a**) and 3D MESP map for molecule PA3N4MP (**b**).

The arrangement of the molecules in the PA3N4MP crystal is mainly determined by electrostatic interactions and van der Waals forces because there are no strong directional interactions (except of intramolecular N–H∙∙∙O hydrogen bond) between the molecules (Figure 2). The inversion-related molecules along the stacks are separated by ~4.70 Å, which indicates a π-π interaction between the π-clouds of the pyridine and phenyl rings that is too weak. However, the electrostatic interactions between the molecules within the stacks, as can be seen from the three-dimensional electrostatic potential map (Figure 2b), take place. The three-dimensional molecular map of the electrostatic potential (3D-MESP) is related with the electron density within the molecule and is helpful in the nucleation and crystal growth processes. The calculated 3D MESP is mapped onto the total electron density isosurface (0.008 eÅ^−3^) and illustrates the size, shape, charge density, and the sites of the chemical reactivity. Arrangement of the neighboring molecules within the stacks as well as between the stacks results from electrostatic interactions between the fragments of molecules with opposite signs of EP (Figure 2b).

#### 2.1.2. Description of the Structure of 2-*N*-Phenylamino-3-nitro-6-methylpyridine

**Figure 3:**
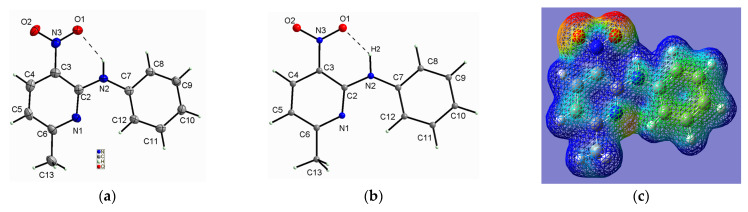
(**a**) View of the X-ray structure of PA3N6MP, (**b**) DFT optimized molecule, and (**c**) 3D EP map; color coded as in Figure 2.

The 2-*N*-phenylamino-3-nitro-6-methylpyridine, PA3N6MP, crystallizes in the centrosymmetric space group *P*2_1_*/n* of the monoclinic system with four molecules per unit cell. Its asymmetric unit contains one 2-phenylamino-3-nitro-6-methylpyridine molecule. Its X-ray molecular structure together with the optimized one, for comparison, is shown in Figure 3, and selected geometrical parameters are listed in Table 2, but full details of the optimized DFT parameters are given in Table S2 (in Supplementary Materials). The conformation of the 2-*N*-phenylamino-3-nitro-6-methylpyridine molecule is not planar. The planar NO_2_ group is slightly twisted in relation to the plane of the pyridine ring by 10.30(12)°, whereas the dihedral angle between the average plane of pyridine ring and the average plane of phenyl ring is 2.90(14)°. A weak intramolecular N–H∙∙∙O is observed. This is reflected in the N–O bond lengths of the NO_2_ group (Table 2). In contrast to the X-ray structure, the gas-phase conformation obtained from DFT calculations shows its strictly planar arrangement. In general, the bond lengths and angles, without that described of the dihedral angles described above, correlate well with that in the crystal.

**Figure 4:**
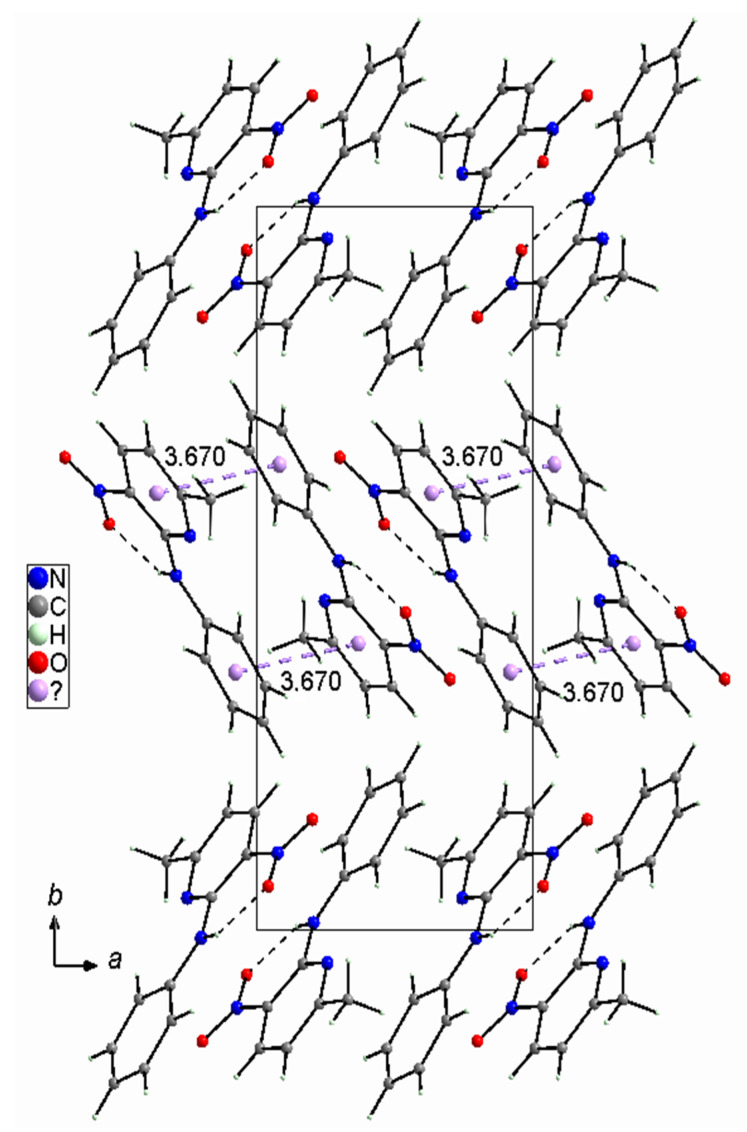
Packing diagram of the PA3N6MP.

Arrangement of the molecules in the crystal are mainly determined by the van der Waals forces and by the stacking interactions along the direction between adjacent molecules (Figure 4). Within the stack there is an alternating arrangement of pyridine and benzene rings with a distance of ~3.670 Å between the centroids of the rings. This alternating arrangement of adjacent molecules is consistent with the 3D MESP, illustrated in Figure 4. This distance indicates quite weak π-π interactions along the stack.

#### 2.1.3. Comparison of the Structures of PA3N4MP and PA3N6MP

**Figure 5:**
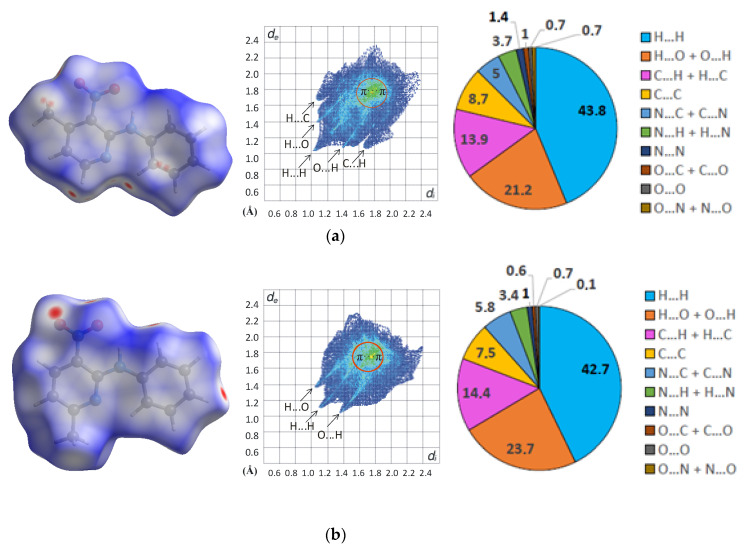
HS (**left**), the 2D-fingerprint plots (**middle**), and the contributions of the respective interactions (**right**) for PA3N4MP (**a**) and PA3N6MP (**b**).

Hirshfeld surface analysis, HS [33], and its corresponding two-dimensional fingerprint plot [34] provide a convenient method for comparing interactions within crystal structures for different isomers, revealing important similarities and differences between them [35,36]. The Hirsfeld surface mapped with *d_borm_* for the molecules of both isomers in the crystals and their 2D fingerprint plots are illustrated in Figure 5.

The normalized contact distance (*d_borm_*) is based on the distances from the nearest atom inside (*d_i_*) and outside (*d_e_*) the surface. In the HS, the de value corresponds to the distance between the external atom and the surface, and di value corresponds to the distance between the internal atom and the surface. The red spots in the HS correspond to the contact distances between the atoms inside and outside the surface, which are smaller than the sum of the van der Waals radii. Blue and white areas represent the distances longer and equal than the sum of the respective van der Waals radii. The deconvolution of the 2D-fingerprint plots for individual types of interactions is given in Figures S1 and S2 (in Supplementary Materials) for PA3N4MP and PA3N6MP, respectively. In the crystal of both isomers (PA3N4MP and PA3N6MP), the main interactions between the molecules are the H···H dispersive forces with the contribution of 43.8% and 42.7% in the HS of PA3N4MP and PA3N6MP, respectively. The second type of interactions between the molecules in the crystals are H···O/O···H, which constitute 21.2% and 23.7%, respectively, in the HS of PA3N4MP and PA3N6MP. The next types of intermolecular interactions in the crystals PA3N4MP and PA3N6MP are C···H/H···C, C···C, and C···N/N···C, which constitute 13.9%, 8.7%, and 5.0% for PA3N4MP and 14.4, 7.5, and 5.8% for PA3N6MP. The contribution of the respective interactions to the HS in the crystals of PA3N4MP and PA3N6MP is illustrated in Figure 5 on the right. In general, the contribution of the individual interactions is quite comparable in the crystals of both isomers, but there is a visible difference between them. In the PA3N4MP crystal, the contribution of the H···O/O···H interactions associated with the hydrogen bonds is about 2.5% smaller than in the crystal of the second isomer, PA3N6MP, while in the PA3N4MP crystal, the contribution of the C···C interactions is 8.7% and is almost 20% larger than in the PA3N6MP crystal, where it is 7.5%.

### 2.2. Vibration Characterization

#### 2.2.1. Vibrational Spectra

**Figure 6:**
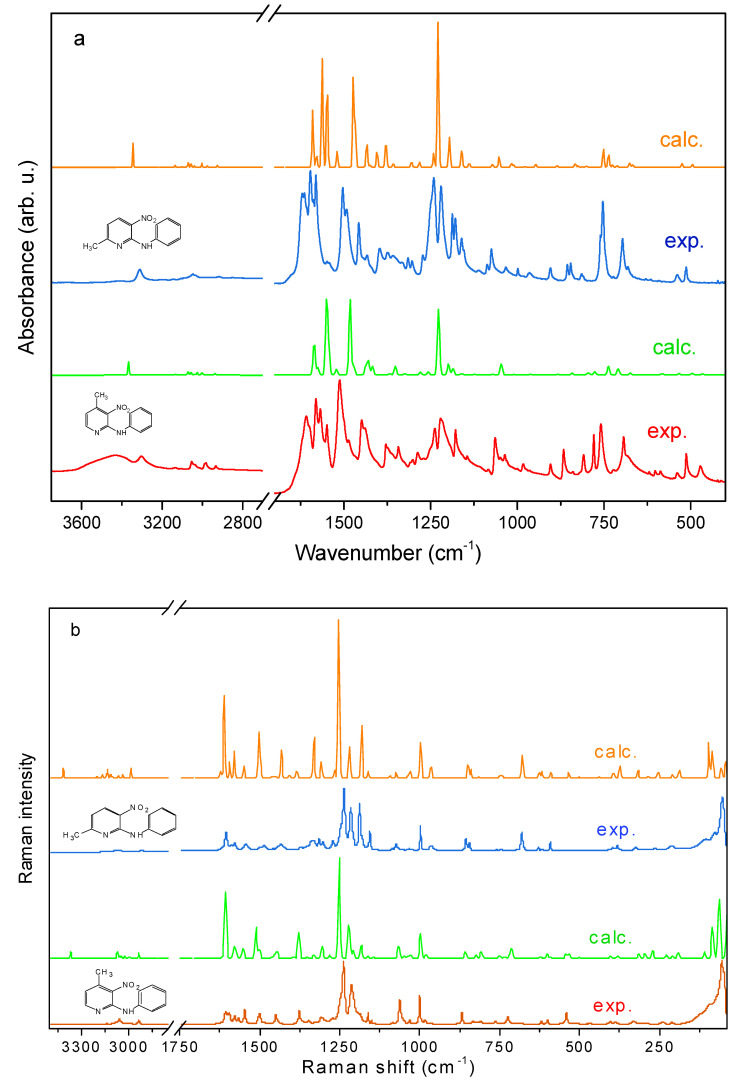
Experimental and calculated IR (**a**) and Raman (**b**) spectra of the studied phenyl–aminopyridines in the range of 3500–100 cm^−1^.

The IR and Raman spectra of the studied compounds are shown in Figure 6.

#### 2.2.2. Vibrational Modes of Pyridine and Phenyl Systems

Given the structural homology between the investigated isomeric systems, characteristic vibrational signatures of the pyridine moiety are anticipated to fall within established spectral ranges for such aromatic frameworks. Vibrational mode assignments were performed using a dual approach: (1) guided by spectral benchmarks from pyridine derivatives documented in the classification schemes of Ureña et al. [37] and Wiberg et al. [38], complemented by comparative analysis of related systems [39,40,41]; (2) quantum chemical modeling of the target molecules.

Computational simulations at the DFT level reveal two distinct categories of vibrational behavior:

- Localized modes arising from discrete atomic displacements, including the following:
  ○ N–H stretching (ν(NH));
  ○ Aromatic C–H stretching (ν(CH));
  ○ Out-of-plane C–H bending (γ(CH));
  ○ Methyl group vibrations (ν(CH_3_;), δ(CH_3_;), ρ(CH_3_;)).
- Collective vibrational modes involving coupled displacements across multiple atomic coordinates. The following ranges are proposed as the pyridine ring vibrations for derivatives PA3N4MP and PA3N6MP (in parenthesis), respectively: ν(CH): 3036–3056, 3130 (3047–3146); ν(ϕ): 1148–1486 (1458–1491); ν(ϕ) + δ(CNH)_ϕ_: 1579–1596 (1580–1596); ν(ϕ) + δ(C_ϕ_NC_Θ_): 1500–1548 (1500–1548); δ(ϕ) and δ(ϕ) + δ(CH): 1238–1286 (1236–1271); δ(CH)_ϕ_: 1048–1063. 810 (1073, 813); τ(ϕ): 600 (592); γ(ϕ): 536–541 (534–536) and δ(ϕ) + ν(Cϕ-NO_2_): 386 (394–395) cm^−1^.

These assignments align with experimental observations and theoretical predictions for π-conjugated systems, confirming the minimal structural perturbation induced by methyl positional isomerism.

The vibrational signatures of the phenyl aromatic system are detected within their characteristic spectral regions [42]. A systematic correlation between the experimentally observed absorption bands and specific normal vibrational modes of the investigated isomeric systems is provided in Table 3.

#### 2.2.3. Methyl Group Vibrational Modes

The methyl substituents, attached at 4- or 6-position of the pyridine ring, exhibit characteristic vibrational features. Mode assignments were guided by comparative DFT analysis and experimental benchmarks from structurally analogous pyridine derivatives [39,40,41,43,44]. Key vibrational parameters for PA3N4MP and PA3N6MP (in brackets) are as follows:

- Asymmetric stretching (ν_as_(CH_3_;)): 2981–3005 cm^−1^ (2980–3006 cm^−1^);
- Symmetric stretching (ν_s_(CH_3_;)): 2932–2935 cm^−1^ (2914–2915 cm^−1^);
- Asymmetric bending (δ_as_(CH_3_;)): 1429–1437 cm^−1^ (1430–1434 cm^−1^);
- Symmetric bending (δ_s_(CH_3_;)): 1326–1278 cm^−1^ (1315–1373 cm^−1^);
- In-plane rocking (ρ(CH_3_;)): 1001–1027 cm^−1^ (1002–1025 cm^−1^);
- Torsional modes (τ(CH_3_;)): ~270 cm^−1^ (264–278 cm^−1^).

These vibrational bands exhibit moderate intensity and frequently overlap with pyridine ring vibrational modes, reflecting coupling between the aromatic system and substituent dynamics.

#### 2.2.4. Nitro Group Vibrations

The nitro substituent (–NO_2_) attached to the pyridine ring exhibits nine fundamental vibrational modes, categorized as follows:

- Stretching vibrations:
  ○ Asymmetric (ν_as_(NO_2_));
  ○ Symmetric (ν_s_(NO_2_));
  ○ C–N linkage stretching (ν(C–NO_2_)).
- In-plane deformations:
  ○ Bending (δ(NO_2_));
  ○ Rocking (ρ(NO_2_));
  ○ Pyridine ring–NO_2_ bending (δ(φ–NO_2_)).
- Out-of-plane motions:
  ○ Wagging (ω(NO_2_));
  ○ Twisting (τ(NO_2_));
  ○ Pyridine ring–NO_2_ torsional (γ(φ–NO_2_))

(where φ denotes the pyridine ring framework)

These vibrational modes demonstrate significant coupling with pyridine ring dynamics, producing hybrid spectral signatures. Nitro group vibrations are characterized by intense IR absorption bands (strong to very strong) and weak Raman activity. Key experimental frequencies for PA3N4MP and PA3N6MP (in parentheses) include the following:

- ν_as_(NO_2_): 1378–1567 cm^−1^ (1434–1578 cm^−1^);
- ν_s_(NO_2_): 1162–1378 cm^−1^ (1156–1373 cm^−1^);
- Scissoring (δ(NO_2_)): 833–845 cm^−1^;
- Wagging (ω(NO_2_)): 720–725 cm^−1^.

Additional in-plane and out-of-plane deformation modes reside within their characteristic frequency ranges, consistent with nitro-functionalized aromatic systems. The observed vibrational coupling underscores the electronic conjugation between the nitro group and the π-electron system of the pyridine ring.

#### 2.2.5. Vibrations of the Amino Bridge

The vibrations of the amino-bridging moiety (-NH-) connecting the phenyl and pyridine rings were analyzed with emphasis on molecular conformation and its role in facilitating intramolecular hydrogen bonding. In the solid-state architecture, the NH group serves as the primary proton donor, critically stabilizing the molecular framework through moderate-strength N–H···O hydrogen bonds involving the nitro group’s oxygen atom.

Spectral features in the 3500–3100 cm^−1^ region are attributed to N–H···O interactions between the amino bridge and the 3-position nitro substituent on the pyridine ring. Distinct N–H stretching modes (ν(NH)) are observed:

- PA3N4MP: 3433 cm^−1^ and 3304 cm^−1^;
- PA3N6MP: 3430 cm^−1^ and 3311 cm^−1^.

These values align closely with reported data for analogous hydrogen-bonded systems [39,40,41]. The bifurcation of ν(NH) bands into doublet components arises from crystallographic packing differences: PA3N4MP contains two symmetry-independent molecules per unit cell, while PA3N6MP exhibits four discrete molecular units in its crystalline lattice. This structural dichotomy directly correlates with the observed splitting magnitude in vibrational spectra.

### 2.3. Electron Reflectance and Emission Spectra

**Figure 7:**
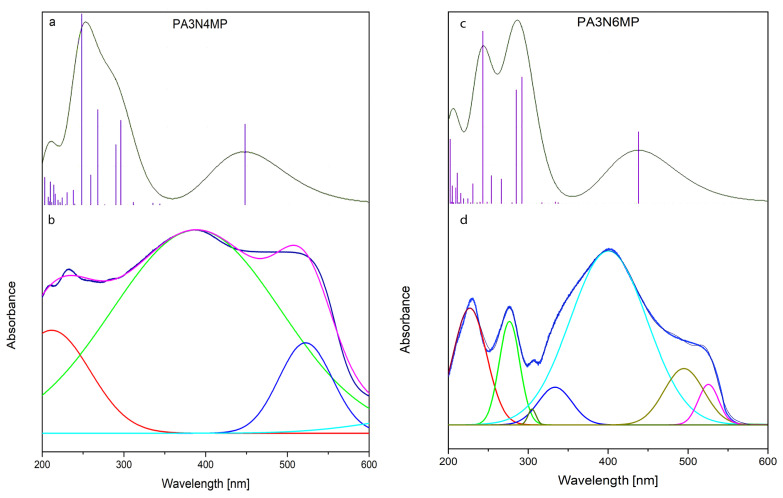
Reflectance UV-Vis spectra of the studied isomers. Calculated: PA3N4MP (**a**), PA3N6MP (**c**). Experimental: PA3N4MP (**b**), PA3N6MP (**d**).

Figure 7 displays the electronic reflectance profiles of the investigated compounds, which are characterized by prominent electronic transitions manifesting as broad absorption contour spanning the 200–600 nm wavelength region. These spectral features were assigned to specific electronic excitations through comparative analysis with prior investigations on structurally related pyridine-based systems [41,42,43,44,45,46], further supported by DFT computational modeling performed for the current derivatives. Table 4 summarizes the theoretically derived energy levels for both singlet and triplet excited states, along with their corresponding oscillator strength values (*f*). Notably, several singlet excitations exhibit exceptionally high oscillator strength values, indicating their pronounced contribution to the observed optical activity. For the PA3N4MP isomer, this appeared for the S_5_ (296 nm, 33,730 cm^−1^, S_6_ (292 nm, 34,260 cm^−1^), S_8_ (267 nm, 37,408 cm^−1^), and S_10_ (251 nm, 39,830 cm^−1^) states observed in the range 100–400 nm, and for the S_1_ state (464 nm, 21,552 cm^−1^) this appeared as the very broad band in the range 200–600. Similar spectroscopic contours appear for the PA3N6MP derivative. The calculated values of the singlet levels appear for the S_5_ (294 nm, 34,056 cm^−1^, S_6_ (288 nm, 34,739 cm^−1^), S_8_ (264 nm, 37,890 cm^−1^), and S_10_ (245 nm, 40,853 cm^−1^) states–they should be assigned to the bands observed in the ranges 250–300 nm and 200–290 nm. The S_1_ state is located at 454 nm (22,031 cm^−1^), which corresponds to the broad band observed in the range 300–500 nm. These spectral contours originate from electronic transitions localized within the π-system of the pyridine ring, as well as charge-transfer interactions between the nitro-chromophore and the aromatic system. These absorption bands correlate with π→π* electronic excitations, which aligns with the calculated energy difference between the HOMO and LUMO orbitals derived from quantum chemical modeling. The atomic composition of the molecular orbitals obtained for the studied compounds is similar to those determined for other amino-methyl-nitro-pyridine derivatives repeated in our earlier works [30,31,32,43,44,45,46]. In the HOMO orbital, the electron distribution is mainly scattered over the *N*-amine group and pyridine C–C and C–N bonds, whereas the LUMO is mainly over the pyridine nitro group. The theoretical HOMO-LUMO energy gap equals 3.13 eV (25,245 cm^−1^, 396 nm) for the PA3N4PT isomer and 3.1617 eV (25,501 cm^−1^, 392 nm) for the PA3N6MP isomer. It fits well to the very strong and broad bands observed in the 200–600 nm range with the maximum at about 400 nm (25,000 cm^−1^)—see Figure 7.

The emission spectra were measured using excitation at 350 nm. This wavelength was chosen because it falls into the 290–450 nm range in which the singlets with greatest oscillator strengths appear. Such procedure allows to recognize the possibility of the triplet→singlet transition phosphorescence in the studied compounds which arises predominantly through intersystem crossing mechanism. Therefore, the complete DFT calculations should give the singlet and triplet transitions in which also are those with zero oscillator strengths. They are important for the discussion of the luminescence spectra activated by the inter-crossing energy transfer process.

**Figure 8:**
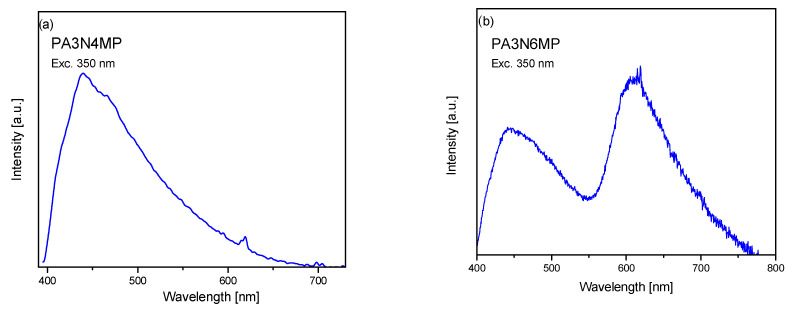
Emission spectra of measured for studied isomers under excitation at 350 nm: (**a**) PA3N4MP, (**b**) PA3N6MP.

The emission spectra of the derivatives studied are shown in Figure 8.

The emission spectra of the studied compounds excited at 350 nm (28,571 cm^−1^) contain a very broad spectral contour observed for PA3N4MP in the range 400–700 nm and two intense bands in the ranges 400–550 and 550–700 nm for PA3N6MP. Their positions are clearly shifted into red in comparison to the observed S_0_ S_m_ absorption transition at 200–600 nm (16,666–50,000 cm^−1^), because the depopulation T_1_,T_2_ S_0_ mechanism of the excited levels was probably proceeded by the inter-system crossing of S_1_ T_1_,T_2_. Room temperature phosphorescence is generally not observed from excited triplet states; however, keeping in mind the calculated energies of the singlet and triplet levels (Table 4), such an explanation of the nature of these transitions is reasonable. The calculated energies of the T_1_ and T_2_ triplet states are localized within the 13,000–14,000 cm^−1^ range, closely matching theoretical predictions for triplet-mediated luminescence in π-conjugated systems. The modest energetic separation between these states (~1000 cm^−1^) likely drives significant spectral overlap, manifesting experimentally as a broad composite T_1_–T_2_ emission band.

This observation agrees with a depopulation mechanism governed by spin–orbit coupling effects, where intersystem crossing (ISC) facilitates efficient energy transfer from singlet to triplet manifolds–a hallmark process in the Jablonski framework for luminescent organic systems. The near-degeneracy of T_1_ and T_2_ energies relative to the ground state (S_0_) results in quasi-simultaneous T_1_→S_0_ and T_2_→S_0_ transitions. Their energetic proximity (ΔE < kT at ambient conditions) prevents discrete spectral resolution, yielding the observed broadened emission profile characteristic of multi-state relaxation pathways.

### 2.4. ^13^C and ^1^H NMR Spectra Measurements

**Figure 9:**
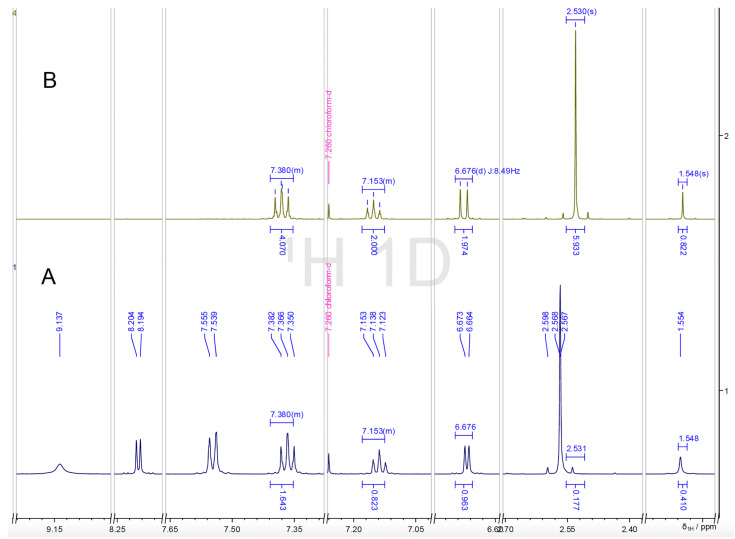
^1^H NMR spectra of the studied isomers: (**A**) 2-*N*-phenylamino-4-methyl-3-nitro- and (**B**) 2-*N*-phenylamino-6-methyl-3-nitro-pyridines.

**Figure 10:**
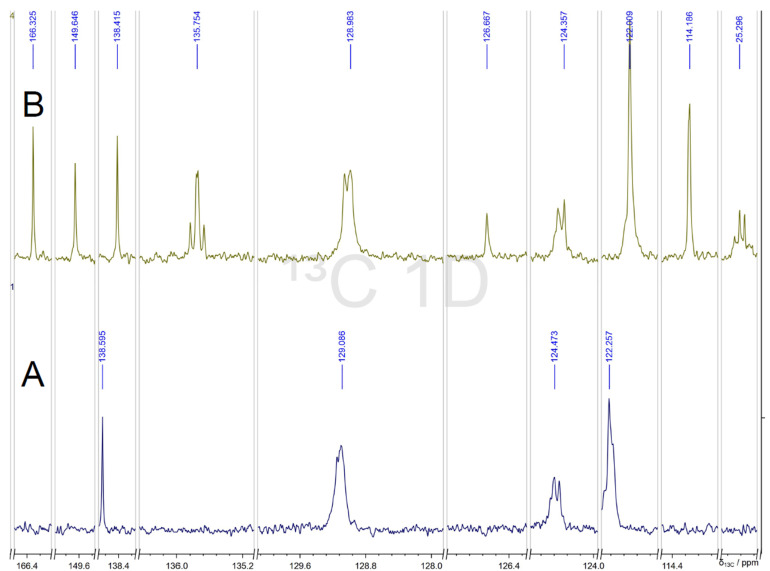
^13^C NMR spectra of the studied isomers: (**A**) 2-*N*-phenylamino-4-methyl-3-nitro- and (**B**) 2-*N*-pheny lamino-6-methyl-3-nitro-pyridines.

The ^1^H and ^13^C NMR spectra of the investigated derivatives are presented in Figure 9 and Figure 10, respectively (full-length spectra are available in the Supplementary Materials—Figure S3 for ^1^H and Figure S4 for ^13^C). The observed spectral pattern–specifically, the number of distinct resonance lines–agrees with predictions derived from the molecular architecture of these systems. Each chemically inequivalent nucleus generates a discrete resonance signal, enabling unambiguous assignment of proton and carbon environments. For the 2-*N*-phenylamino-4-methyl-3-nitro-, and 2-*N*-phenylamino-6-methyl-3-nitro-pyridines, ^13^C chemical shifts are expected for the carbon atoms constituting the pyridine ring, phenyl ring, and one from the methyl group.

The molecular composition of the studied isomers predicts ten distinct ^13^C NMR resonances:

- Pyridine ring: five unique carbons;
- methyl group: one resonance;
- phenyl ring: four signals (with two symmetry-equivalent carbon pairs).

Experimental ^13^C NMR spectra for both PA3N4MP and PA3N6MP isomers corroborate this theoretical framework, exhibiting ten resolved signals that map directly to their respective chemical environments. This agreement validates the proposed structural symmetry and atomic degeneracy within the aromatic systems.

For PA3N4MP, the chemical shifts in the pyridine ring are observed at 151.76, 150.08, 131.92, 122.26, and 118.13 ppm but for PA3N6MP at 166.33, 149.664, 128.98, 122.01, and 140.19 ppm. The chemical shifts in the phenyl carbon atoms appear at 146.45, 138.60, 129.09, and 124.47 ppm for the PA3N4MP isomer and 138.41, 135.75, 126.67, and 124.36 ppm for the PA3N6MP isomer. The line at 21.51 ppm corresponds to the carbon atom of the methyl chromophore in the PA3N4MP derivative and at 25.30 ppm in the PA3N6NP isomer. These carbon atoms are located in the vicinity of three hydrogen atoms and, therefore, show the greatest chemical shifts.

Similar analysis can be carried out for the proton units. In the ^1^H NMR spectra of the studied isomers, nine lines are expected: two from the pyridine ring, five from the phenyl ring, one from the methyl group, and one of the NH chromophore. They are observed at the following chemical shifts: at 7.55, 7.37, 7.14, 6.85, and 6.67 ppm originated from the phenyl protons of the PA3N4MP isomer and at 7.73, 7.31, 7.15, 6.85, and 6.68 ppm in the PA3N6MP isomer. The chemical shifts in the pyridine protons appear at 9.14 and 8.20 ppm for PA3N4MP and at 10.25 and 8.41 ppm for PA3N6MP. The protons of the NH chromophores give rise to the peaks at 5.93 and 5.93 ppm for the PA3N4MP and PA3N6MP isomers, respectively, and those of methyl groups at 1.56 and 1.55 ppm.

Proposed here, assignment of the chemical shifts observed for the studied derivatives fit well to the reported earlier data reported for the similar compounds [47,48,49,50].

## 3. Materials and Methods

### 3.1. Synthesis

Two isomers:

- 2-*N*-phenylamino-3-nitro-4-methylpyridine
- 2-*N*-phenylamino-3-nitro-6-methylpyridine

The two isomers were synthesized following a general procedure inspired by the methodology outlined in [51]. However, due to the limited accessibility of this reference–it is neither available online nor widely distributed–an overview of the synthetic approach is provided here in the form of reaction schemes to aid reproducibility and comprehension.

**Scheme 1:**
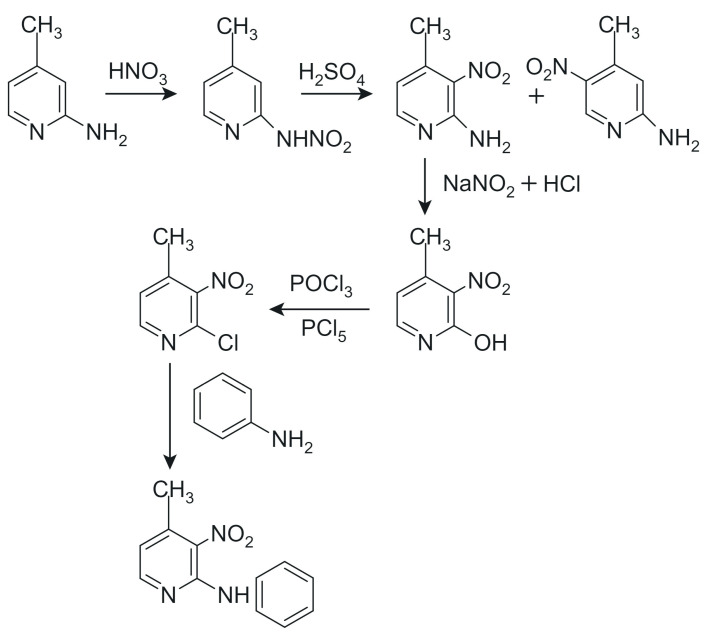
Synthesis of 2-*N*-phenylamino-3-nitro-4-methylpyridine.

**Scheme 2:**
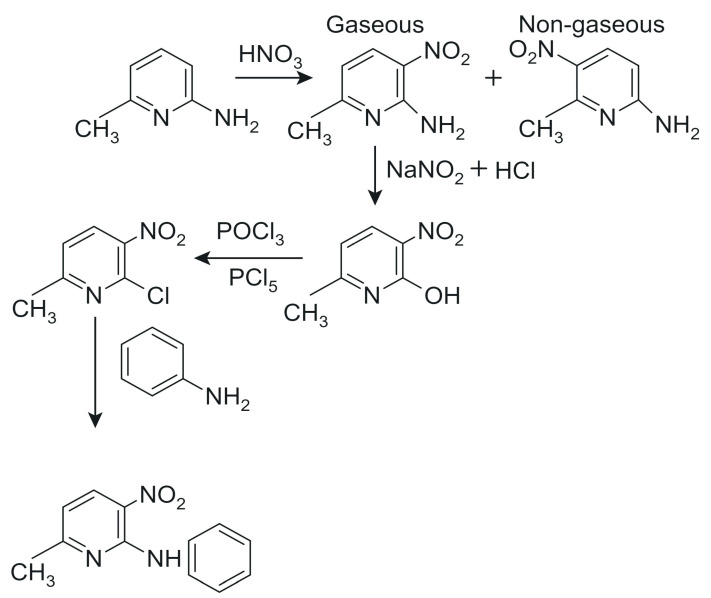
Synthesis of 2-*N*-phenylamino-3-nitro-6-methylpyridine.

The synthesis began with 2-amino-4-methylpyridine and 2-amino-6-methylpyridine, which were nitrated and subsequently converted into 2-hydroxy derivatives. These intermediates were then treated with phosphoryl chloride (POCl3) to yield the corresponding 2-chloro derivatives, which served as substrates for the final reaction with phenylamine to form the target compounds. The final products were purified via multiple recrystallizations from ethanol solutions to ensure high purity. Synthesis schemes are presented in Scheme 1 and Scheme 2.

The synthesis began with 2-amino-4-methylpyridine and 2-amino-6-methylpyridine, which were nitrated and subsequently converted into 2-hydroxy derivatives. These intermediates were then treated with phosphoryl chloride (POCl3) to yield the corresponding 2-chloro derivatives, which served as substrates for the final reaction with phenylamine to form the target compounds. The final products were purified via multiple recrystallizations from ethanol solutions to ensure high purity.

The chemical composition of the synthesized compounds was verified through microanalysis, which confirmed a good agreement with theoretical stoichiometry. The following are the chemical names, formulas, and abbreviations used in this study:

- 2-*N*-phenylamino-3-nitro-4-methylpyridine
  Chemical formula: C_12_H_11_N_3_O_2_
  Abbreviation: PA3N4MP
  Description: Red crystals
- 2-*N*-phenylamino-3-nitro-6-methylpyridine
  Chemical formula: C_12_H_11_N_3_O_2_
  Abbreviation: PA3N6MP
  Description: Red crystals
  Elemental chemical analysis:
- PA3N4MP (red crystals):
  ○ Calculated: C 62.87%, H 4.84%, N 18.33%
  ○ Experimental: C 62.51%, H 4.79%, N 18.17%
  ○ Melting point: 395 K, 122 °C
  ○ Synthetic yield: 79.0%
- PA3N6MP (red crystals):
  ○ Calculated: C 62.87%, H 4.84%, N 18.33%
  ○ Experimental: C 62.55%, H 4.88%, N 18.12%
  ○ Melting point: 405 K, 132 °C
  ○ Synthetic yield: 71.0%

The inclusion of reaction schemes in this work ensures that the general synthetic route, as adapted from [51], is clearly understood and reproducible despite the restricted availability of the original reference. These schemes highlight the key transformations and provide a concise summary of the methodology employed.

**Figure 11:**
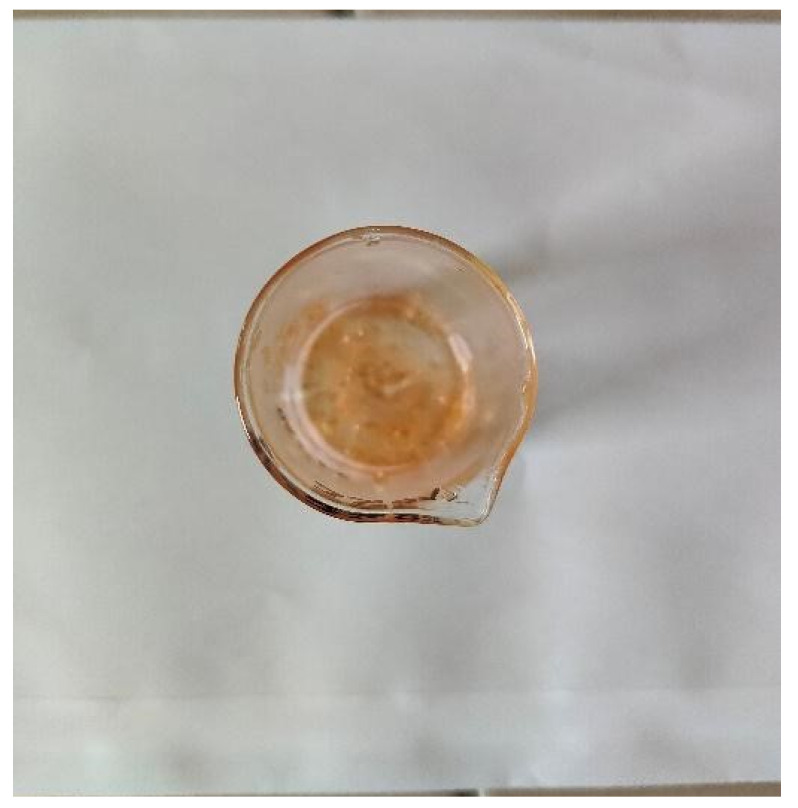
Chitosan gel dyed with the isomer 2-*N*-phenylamino-3-nitro-6-methylpyridine.

The Figure 11 illustrates the uniform coloration of the gel, demonstrating the 2-*N*-phenylamino-3-nitro-6-methylpyridine compound excellent solubility and color intensity, confirming its potential application in the production of packaging films.

### 3.2. Single-Crystal X-Ray Diffraction 128–142

Single crystals of PA3N4MP and PA3N6MP suitable for diffraction studies were grown through slow solvent diffusion using a methanol/ethanol mixed system. X-ray intensity data for both compounds were acquired on a κ-geometry Xcalibur diffractometer equipped with a Sapphire2 CCD detector, employing graphite-monochromated Mo Kα radiation (λ = 0.71073 Å). Data acquisition and initial processing, including frame integration and absorption corrections, were executed using the CrysAlis CCD and CrysAlis RED ver. 1.171.35.11 software suite [52].

Structural solutions were derived via direct methods (SHELXT-2014/7 [53]), followed by iterative full-matrix least-squares refinement against *F*^2^ values (SHELXL-2018/3 [54]). Hydrogen atoms bonded to carbon were geometrically positioned and constrained using a riding model, while those participating in hydrogen bonds underwent unrestrained refinement. Post-refinement difference Fourier analysis revealed no residual electron density features exceeding 0.3 eÅ^−3^, confirming structural validity.

Critical experimental parameters, crystallographic metrics, and refinement statistics are tabulated in Table 5. Molecular topology representations were generated using Diamond 3.0 [55], with thermal ellipsoids plotted at 50% probability.

Hirshfeld surface analyses and 2D fingerprint plots as well as percentage contributions for various intermolecular contacts in the investigated crystals were calculated using the Crystal Explorer Ver. 3.1 program package [56].

### 3.3. Infrared and Raman Studies

IR spectra were measured using a Nicolet iS50 FT-IR (Thermo Fisher Scientific Inc., Warsaw, Poland) spectrometer equipped with an Automated Beamsplitter exchange system (iS50 ABX containing DLaTGS KBr detector (Thermo Fisher Scientific Inc., Warsaw, Poland) and DLaTGS Solid Substrate detector (Thermo Fisher Scientific Inc., Warsaw, Poland) for mid-IR and far-IR regions, respectively). A built-in all-reflective diamond ATR module (iS50 ATR), Thermo Scientific Polaris^TM^ (Thermo Fisher Scientific Inc., Warsaw, Poland), and a HeNe laser were used as an IR radiation source. Polycrystalline IR spectra were collected in the 4000–100 cm^−1^ range. The advanced ATR correction software, part of the OMNIC^TM^ 6.2 program attached to the Nicolet^TM^ FT-IR spectrometer (Thermo Fisher Scientific Inc., Warsaw, Poland), was used in the studies of polycrystalline samples. A spectral resolution of 4 cm^−1^ was applied in the measurements.

Raman spectra in the 4000–80 cm^−1^ range were measured in backscattering geometry with a FT Bruker 110/S spectrometer (Bruker, Warsaw, Poland). The resolution was 2.0 cm^−1^. The YAG:Nd (excitation wavelength 1064 nm) laser was used as an excitation source.

### 3.4. Electronic Absorption Spectroscopy

Electronic absorption spectra in 200–1500 nm spectral range were measured at ambient temperature using a Cary Varian 5E UV-Vis-NIR spectrophotometer (SpectraLab Scientific Inc., Warsaw, Poland). For samples exhibiting low absorption intensity, diffuse reflectance spectroscopy was employed with a Praying Mantis accessory (Harrick Scientific Products Inc., Warsaw, Poland). Prior to sample measurements, a baseline correction was performed using high-purity Al_2_O_3_ powder as a reference standard, which was subsequently subtracted from the spectra to isolate specific absorption features, characteristic for the studied isomers.

### 3.5. Time-Resolved Emission Spectroscopy

Time-dependent emission profiles were determined using a Princeton Instruments Acton 2500i spectrograph interfaced with a Hamamatsu C5680 streak camera (HAMAMATSU Co., Ltd., Warsaw, Poland). Excitation of the spectra was achieved using a femtosecond Ti:sapphire laser (Coherent, Inc., Warsaw, Poland), generating 89 fs pulses at 800 nm (1 mJ/pulse, repetition rate adjustable up to 1 kHz). Wavelength tuning across the 230–2800 nm range was accomplished using an optical OPerA Solo parametric amplifier (Coherent Inc., Warsaw, Poland), enabling selective excitation at targeted energies.

### 3.6. Computational Methodology

Initial molecular geometries for quantum chemical simulations were derived from X-ray crystallographic data using Gaussian 03 [57]. Structural optimizations were executed at the B3LYP/6-311G(2d,2p) level of theory [58,59,60,61,62], yielding a single conformer with minimized vibrational energy. To facilitate comparison with prior studies on analogous pyridine derivatives [30,31,32], harmonic vibrational frequencies were scaled by empirical factors (Table 3, Table S3) compensating for anharmonicity and theoretical approximations.

Potential energy distribution (PED) analyses were performed using the BALGA algorithm [63], while molecular orbital visualizations (HOMO/LUMO) and vibrational mode animations were generated using GaussView 4.1 [64] and ChemCraft ver. 1.8 [65], respectively. Raman intensities were computed with the RAINT program ver. 2003 [66], employing a 0.965 scaling coefficient derived from established protocols [67].

### 3.7. NMR Spectroscopy

^13^C and ^1^H nuclear magnetic resonance spectra were acquired on a Bruker Avance III 600 MHz spectrometer fitted with a BBO Z-gradient probe. Liquid-state measurements utilized a 3.2 mm magic-angle spinning (MAS) probe ((Bruker, Warsaw, Poland)), optimizing resolution through high-speed sample rotation.

## 4. Conclusions

- In the present work, two new heterocyclic isomers, 2-*N*-phenylamino-4-methyl-3-nitro- and 2-*N*-phenylamino-6-methyl-3-nitro-pyridines, were obtained. Their structures were characterized on the basis of X-ray diffraction, IR and Raman spectra, as well as electron UV-Vis and emission spectra measurements. They form new class of pyridine derivatives with several potential applications. This follows from their peculiar structural, electronic, and optical properties.
- The specific structure of the studied compounds originates from the amino bridge between the phenyl and pyridine rings, and this conformation is stabilized by the intramolecular medium-strength hydrogen bond of N–H···O type formed by the O-atom of the nitro group and the NH group. The NH group plays an important role as the proton donor in the structural stabilization of the studied compounds in the solid state. Such structure predisposes these derivatives as precursors of antihistamine drugs, which can be used to oppose the activity of histamine receptors in the human body, forming antihistaminic pharmaceutics for allergic disorders. Such drugs contain the pyridine ring as an important part of their structure [68].
- Integrated spectroscopic analysis and DFT computational modeling reveal distinct singlet-triplet state distributions governing the photophysical behavior of the investigated pyridine isomers. Key findings include the following:
  ○ Phosphorescence mechanism: electronic absorption and emission spectral data confirm that triplet→singlet phosphorescence arises predominantly through intersystem crossing machanism (ISC), a well-documented relaxation pathway in nitro-functionalized pyridine systems.
  ○ Structure–function correlation: unique electronic architecture of phenyl-aminopyridine derivatives enables their dual functionality as follows:
    ■ Versatile synthetic building blocks for heterocyclic chemistry;
    ■ Efficient ligands for d-/f-block metal coordination complexes;
    ■ Tunable luminophores with tailorable ISC efficiency.
  ○ Technological potential: engineered lanthanide complexes incorporating these scaffolds demonstrate promise as follows:
    ■ Targeted bioimaging probes leveraging metal-centered luminescence;
    ■ Photosensitizers for photodynamic therapy applications;
    ■ Molecular sensors for environmental metal ion detection.
- The ^13^C and ^1^H NMR spectra fully confirm the structural XRD data reported in the present work.
- The intense red color of the obtained materials allow for their use as new dyes for the production of the color plastic foils for food packing.
- This work establishes foundational structure–property relationships critical for rational design of next-generation luminescent materials in medicinal and materials chemistry.

## Figures and Tables

**Table 1:** Selected geometrical parameters (Å, °) for PA3N4MP.

			X-Ray			DFT	
N3–O1			1.2412(15)			1.242	
N3–O2			1.2272 (14)			1.226	
C3–N3			1.4508 (16)			1.454	
C2–N2–C7			131.01 (11)			131.64	
Dihedral angle between the planes:							
NO_2_ (O1N3O2)/pyridine ring (N1,C2–C6)			3.84(15)			25.50	
pyridine ring (N1,C2–C6)/phenyl ring (C7–C12)			6.20(15)			15.70	
D–H···A	D–H	H···A		D···A	D–H···A		
N2–H2···O1	0.900(16)	1.860(15)		2.5877(14)	136.4(13)		X-ray
N2–H2···O1	1.014	1.814		2.632	135.17		DFT

**Table 2:** Selected geometrical parameters (Å, °) for PA3N6MP.

		X-Ray				DFT				
N3–O1		1.249 (2)				1.243				
N3–O2		1.230 (2)				1.226				
C3–N3		1.447 (3)				1.450				
C2–N2–C7		132.0 (2)				132.24				
Dihedral angle between the planes:										
NO_2_ (O1N3O2)/pyridine ring (N1,C2–C6)		3.84 (15)				0.00				
pyridine ring (N1,C2–C6)/phenyl ring (C7–C12)		6.20 (15)				0.00				
D–H···A		D–H		H···A				D···A		D–H···A
N2–H2···O1	0.84 (2)		1.96 (2)		2.649 (3)		139 (2)		X-ray	
N2–H2···O1	1.016		1.816		2.649		136.58		DFT	

**Table 3:** Chosen experimental and calculated wavenumbers with assignment of the observed bands to respective normal modes for monomer.

PA3N4MP				PA3N6MP				
Calc.		Exp.		Calc.		Exp.		
IR	RS	IR	RS	IR	RS	IR	RS	Assignment
3367	3367	3433m		3344	3344	3311w		νN–H····O
1585	1585	1579s	1579w	1589	1589	1580vs	1585w	δ(CNH)_ϕ_ + ν(ϕ)
1574	1574	1566s	1567w	1578	1578		1578w	δ(CNH)_θ_ + ν(ϕ) + ν_as_(NO_2_)
1549	1549	1548s	1547m	1548	1548	1548vw	1545w	ν_as_(NO_2_) + ν(ϕ) + ν(θ) + δ(C_ϕ_ NC_θ_)
1350	1350	1361		1357	1357	1357w		ν(CN)_ϕ_ + ν_s_(NO_2_) + δ(ϕ) + δ_s_(CH_3_)
1226	1226	1237m	1212s	1227	1227	1219vs	1216s	ν(ϕ) + ν_s_(NO) + ν(CN)_ϕα_ + ν(CNH)_ϕ_
1197	1197	1220s	1192sh	1195	1195	1186s	1188s	ν_s_(NO_2_) + (CNH)_θ_
1184	1184	1203sh						ν_s_(NO_2_) + ν(NCN)_ϕ_
807	807	807m						ν(CNHC)
790	790							ν(CNHC)_ϕ+θ_ + δ_s_(NO)_ϕ_
707	707			710	710			δ(NH)
699	699	691m				694m		δ(CNHC)_ϕ+θ_
395	395		386w	386	386	394w	395vw	δ(CCH_3_) + δ(CNO)_ϕ_
290	290		289w			295m	295vw	δ(CNHC)_ϕ+θ_
187	187			184	184	188w		δ(CNHC)_ϕ+θ_
107	107			94	94	177w		δ(CNHC)_ϕ+θ_
83	83			84	84	150m		δ_s_(NO)_ϕ_ + δ(CNHC)_ϕ+θ_

**Table 4:** Calculated singlet and triplet electron levels of the PA3N4MP (a) and PA3N6MP (b) molecules.

(a)				
Electron levels	eV	nm	cm^−1^	Oscillator strength
**singlets**				
(1)	2.6717	464.06	21,552	0.1230
(2)	2.7783	446.25	22,409	0.1648
(3)	3.6225	342.26	29,218	0.0008
(4)	3.6351	341.07	29,319	0.0009
(5)	3.7309	332.32	30,092	0.0022
(6)	3.7385	331.64	30,153	0.0026
(7)	4.0091	309.26	32,336	0.0038
(8)	4.1820	296.47	33,730	0.1643
(9)	4.2399	292.42	34,197	0.1730
(10)	4.2476	291.89	34,260	0.0935
(11)	4.3350	286.00	34,965	0.1234
(12)	4.5328	273.53	36,559	0.0007
(13)	4.6380	267.32	37,408	0.1533
(14)	4.8088	257.83	38,785	0.0237
(15)	4.9383	251.07	39,830	0.2944
(16)	5.0938	243.40	41,085	0.3909
(17)	5.2701	235.26	42,506	0.0019
(18)	5.3092	233.53	42,821	0.0295
(19)	5.5492	223.43	44,757	0.0007
(20)	5.8961	210.28	47,556	0.0212
**triplets**				
(1)	1.6711	741.93	13,478	0.0000
(2)	1.7361	714.16	14,002	0.0000
(3)	1.8193	681.49	14,674	0.0000
(4)	1.9895	623.19	16,046	0.0000
(5)	2.1489	576.96	17,332	0.0000
(6)	2.7649	448.42	22,300	0.0000
(b)				
Electron levels	eV	nm	cm^−1^	Oscillator strength
**singlets**				
(1)	2.7315	453.91	22,031	0.1285
(2)	2.8418	436.28	22,921	0.1693
(3)	3.7076	334.40	29,904	0.0000
(4)	3.7191	333.37	29,997	0.0001
(5)	3.7537	330.30	30,030	0.0007
(6)	3.7585	329.88	30,314	0.0010
(7)	3.9573	313.31	31,917	0.0000
(8)	3.9688	312.40	32,010	0.0001
(9)	4.2224	293.63	34,056	0.2176
(10)	4.3072	287.86	34,739	0.2413
(11)	4.5014	275.43	36,307	0.0007
(12)	4.6978	263.92	37,890	0.0396
(13)	4.9311	251.43	39,773	0.0334
(14)	5.0652	244.78	40,853	0.2846
(15)	5.2259	237.25	42,150	0.4088
(16)	5.3044	233.74	42,783	0.0018
(17)	5.5145	224.83	44,478	0.0449
(18)	5.6807	218.25	45,819	0.0095
(19)	5.8055	213.56	46,825	0.0101
(20)	5.9308	209.05	47,835	0.0233
**triplets**				
(1)	1.6560	748.71	13,356	0.0000
(2)	1.7222	719.90	13,891	0.0000
(3)	1.7516	707.82	14,128	0.0000
(4)	1.9444	637.65	15,683	0.0000
(5)	2.0811	595.77	16,785	0.0000
(6)	2.2669	546.94	18,284	0.0000
(7)	2.5069	494.58	20,219	0.0000
(8)	2.5736	481.76	20,757	0.0000
(9)	2.8381	436.86	22,891	0.0000
(10)	3.0912	401.08	24,933	0.0000

**Table 5:** Crystal data and refinement parameters for the studied crystals.

	PA3N4MP	PA3N6MP
Formula	C_12_H_11_N_3_O_2_	C_12_H_11_N_3_O_2_
Mol. weight	229.24	229.24
Temperature (K)	100(2)	100(2)
Crystal system	triclinic	monoclinic
Crystal color	red	red
Space group	*P*-1	*P*2_1_*/n*
*a* (Å)	7.3285(5)	7.8874(5)
*b* (Å)	7.4403(6)	17.3681(11)
*c* (Å)	10.9340(11)	8.4396(7)
α (°)	101.781(8)	
β (°)	102.348(7)	111.309(9)
γ (°)	109.981(7)	
*V* (Å^3^)	521.79(8)	1077.09(15)
Z	2	4
*D*_cal_	1.459	1.414
θ range (°)	3.056–29.312	2.844–29.331
μ (mm^−1^)	0.103	0.100
Crystal size	0.32 × 0.24 × 0.13	0.27 × 0.09 × 0.08
T_min._/T_max._	0.9825/1.000	0.9792/1.000
Total/unique/obs. refls.	4590/2420/1955	7173/2565/1515
*R_int_*	0.0158	0.0545
*R* [*F^2^ > 2σ(F^2^)*] *^a^*	0.0414	0.0650
*wR* [*F^2^* all refls] *^a^*	0.1129	0.1359
*S*	0.978	1.001
Δρ_max_, Δρ_min_ (eÅ^−3^)	+0.228; −0.324	+0.244; −0.247

*^a^ R* = Σ ||*F*_o_|–|*F*_c_||/Σ*F*_o_, *wR* = {Σ [*w*(*F*_o_^2^–*F*_c_^2^)^2^]/Σ*wF*_o_^4^}^½^; *w*^−1^ = σ^2^(*F*_o_^2^) + (*aP*)^2^ + *bP* where *P* = (*F*_o_^2^ + 2*F*_c_^2^)/3, and the *a* = 0.0578 and *b* = 0.1467 for crystal 1, *a* = 0.0424 and *b* = 0.0 for crystal 2; *a* = 0.0333 and *b* = 1.2084 for crystal 3 and *a* = 0.0481 and *b* = 0.1271 for crystal 4.

## Data Availability

The data presented in this study are available on request from the corresponding author.

## Supplementary Materials

The following supporting information can be downloaded at: https://www.mdpi.com/article/10.3390/ijms26072874/s1.

## References

[1] El-Gazzar A.-R., Hussein H., Hafez H. (2007). Synthesis and biological evaluation of thieno [2,3-d]pyrimidine derivatives for anti-inflammatory, analgesic and ulcerogenic activity. Acta Pharm..

[2] Hsu M.-H., Savas Ü., Lasker J.M., Johnson E.F. (2011). Genistein, Resveratrol, and 5-Aminoimidazole-4-carboxamide-1-β-d-ribofuranoside Induce Cytochrome P450 4F2 Expression through an AMP-Activated Protein Kinase-Dependent Pathway. J. Pharmacol. Exp. Ther..

[3] Bernardino A.M.R., de Azevedo A.R., Pinheiro L.C.d.S., Borges J.C., Carvalho V.L., Miranda M.D., de Meneses M.D.F., Nascimento M., Ferreira D., Rebello M.A. (2007). Synthesis and antiviral activity of new 4-(phenylamino)/4-[(methylpyridin-2-yl)amino]-1-phenyl-1H-pyrazolo[3,4-b]pyridine-4-carboxylic acids derivatives. Med. Chem. Res..

[4] Wang N.-Y., Zuo W.-Q., Xu Y., Gao C., Zeng X.-X., Zhang L.-D., You X.-Y., Peng C.-T., Shen Y., Yang S.-Y. (2014). Discovery and structure–activity relationships study of novel thieno[2,3-b]pyridine analogues as hepatitis C virus inhibitors. Bioorganic Med. Chem. Lett..

[5] Elansary A.K., Moneer A.A., Kadry H.H., Gedawy E.M. (2012). Synthesis and anticancer activity of some novel fused pyridine ring system. Arch. Pharmacal Res..

[6] Lauria A., Abbate I., Patella C., Martorana A., Dattolo G., Almerico A.M. (2013). New annelated thieno[2,3-e][1,2,3]triazolo[1,5-a]pyrimidines, with potent anticancer activity, designed through VLAK protocol. Eur. J. Med. Chem..

[7] Mohareb R.M., Al-Omran F., Azzam R.A. (2014). Heterocyclic ring extension of estrone: Synthesis and cytotoxicity of fused pyran, pyrimidine and thiazole derivatives. Steroids.

[8] Nikkhoo A.R., Miri R., Arianpour N., Firuzi O., Ebadi A., Salarian A.A. (2013). Cytotoxic activity assessment and c-Src tyrosine kinase docking simulation of thieno[2,3-b] pyridine-based derivatives. Med. Chem. Res..

[9] Ahmed S.A., Ahmed O.M., Elgendy H.S. (2014). Novel synthesis of puriensanalougues and thieno [2, 3-b] pyridine derivatives with anticancer and antioxidant activity. J. Pharm. Res..

[10] Bakhite E.A., Abdel-Rahman A.E., Mohamed O.S., Thabet E.A. (2000). Synthesis, reactions and antimicrobial activity of new cyclopenta [e] thieno [2, 3-b] pyridines and related heterocyclic systems. Pharmazie.

[11] Altalbawy F.M.A. (2013). Synthesis and Antimicrobial Evaluation of Some Novel Bis-α,β-Unsaturated Ketones, Nicotinonitrile, 1,2-Dihydropyridine-3-carbonitrile, Fused Thieno [2,3-*b*]pyridine and Pyrazolo [3,4-*b*]pyridine Derivatives. Int. J. Mol. Sci..

[12] Bumpus N.N., Johnson E.F. (2011). 5-Aminoimidazole-4-carboxyamide-ribonucleoside (AICAR)-Stimulated Hepatic Expression of Cyp4a10, Cyp4a14, Cyp4a31, and Other Peroxisome Proliferator-Activated Receptor α-Responsive Mouse Genes Is AICAR 5′-Monophosphate-Dependent and AMP-Activated Protein Kinase-Independent. J. Pharmacol. Exp. Ther..

[13] Peyton K.J., Liu X.-M., Yu Y., Yates B., Durante W. (2012). Activation of AMP-Activated Protein Kinase Inhibits the Proliferation of Human Endothelial Cells. J. Pharmacol. Exp. Ther..

[14] Nakajima K., Komiyama Y., Hojo H., Ohba S., Yano F., Nishikawa N., Aburatani H., Takato T., Chung U.-I. (2010). Enhancement of bone formation ex vivo and in vivo by a helioxanthin-derivative. Biochem. Biophys. Res. Commun..

[15] Maeda Y., Hojo H., Shimohata N., Choi S., Yamamoto K., Takato T., Chung U.-I., Ohba S. (2013). Bone healing by sterilizable calcium phosphate tetrapods eluting osteogenic molecules. Biomaterials.

[16] Gemba T., Ninomiya M., Matsunaga K., Ueda M.J. (1993). Effects of a novel calcium antagonist, S-(+)-methyl-4, 7-dihydro-3-isobutyl-6-methyl-4-(3-nitrophenyl) thieno [2, 3-b] pyridine-5-carboxylate (S-312-d), on ischemic amino acid release and neuronal injury in stroke-prone spontaneously hypertensive rats. Pharmacol. Exp. Ther..

[17] Balzarini J., Stevens M., Andrei G., Snoeck R., Strunk R., Pierce J.B., Lacadie J.A., De Clercq E., Pannecouque C. (2002). Pridine oxide derivatives: Structure-activity relationship for inhibition of human immunodeficiency virus and cytomegalovirus replication in cell culture. Helv. Chim. Acta.

[18] Stevens M., Pannecouque C., De Clercq E., Balzarini J. (2003). Inhibition of human immunodeficiency virus by a new class of pyridine oxide derivatives. Antimicrob. Agents Chemother..

[19] Stevens M., Pannecouque C., De Clercq E., Balzarini J. (2003). Novel human immunodeficiency virus (HIV) inhibitors that have a dual mode of anti-HIV action. Antimicrob. Agents Chemother..

[20] Balzarini J., Stevens M., De Clercq E., Schols D., Pannecouque C. (2005). Pyridine N-oxide derivatives: Unusual anti-HIV compounds with multiple mechanisms of antiviral action. J. Antimicrob. Chemother..

[21] Scriven E.F.V., Murugan R. (2005). Pyridine and pyridine derivatives. Kirk-Othmer Encyclopedia of Chemical Technology.

[22] Liu C., Luo J., Xu L., Huo Z. (2013). Synthesis of 2-substituted pyridines from pyridine N-oxides. Arkivoc.

[23] Dzierszinski F., Coppin A., Mortuaire M., Dewailly E., Slomianny C., Ameisen J.-C., DeBels F., Tomavo S. (2002). Ligands of the Peripheral Benzodiazepine Receptor Are Potent Inhibitors of *Plasmodium falciparum* and *Toxoplasma gondii* In Vitro. Antimicrob. Agents Chemother..

[24] Dressler H., Considine D.M., Considine G.D. (2006). Pyridine and derivatives. Van Nostrand’s Scientific Encyclopedia Van Nostrand’s Scientific Encyclopedia.

[25] Balzarini J., Keyaerts E., Vijgen L., Vandermeer F., Stevens M., De Clercq E., Egberink H., Van Ranst M. (2005). Pyridine N-oxide derivatives are inhibitory to the human SARS and feline infectious peritonitis coronavirus in cell culture. J. Antimicrob. Chemother..

[26] Youssif S. (2001). Recent trends in the chemistry of pyridine N-oxides. Arkivoc.

[27] Kilenyi S.N., Mousseau J.J. (2015). Pyridine N-oxide. Encyclopedia of Reagents for Organic Synthesis.

[28] Carlin R.L. (1961). Transition Metal Complexes of Pyridine N-Oxide. J. Am. Chem. Soc..

[29] Sarma R., Karmakar A., Baruah J.B. (2008). Synthesis and characterization of pyridine N-oxide complexes of manganese, copper and zinc. Inorganica Chim. Acta.

[30] Lorenc J. (2012). Dimeric structure and hydrogen bonds in 2-*N*-ethylamino-5-metyl-4-nitro-pyridine studied by XRD, IR and Raman methods and DFT calculations. Vib. Spectrosc..

[31] Szatyłowicz H. (2008). Structural aspects of the intermolecular hydrogen bond strength: H-bonded complexes of aniline, phenol and pyridine derivatives. J. Phys. Org. Chem..

[32] Bryndal I., Kucharska E., Wandas M., Lorenc J., Hermanowicz K., Mączka M., Lis T., Marchewka M., Hanuza J. (2014). Comprehensive physicochemical studies of a new hybrid material: 2-Amino-4-methyl-3-nitropyridinium hydrogen oxalate. Spectrochim. Acta A.

[33] Spackman M.A., Jayatilaka D. (2009). Hirshfeld surface analysis. CrystEngComm.

[34] McKinnon J.J., Spackman M.A., Mitchell A.S. (2004). Novel tools for visualizing and exploring intermolecular interactions in molecular crystals. Acta Crystallogr. Sect. B Struct. Sci..

[35] McKinnon J.J., Mitchell A.S., Spackman M.A. (1998). Hirshfeld Surfaces: A New Tool for Visualising and Exploring Molecular Crystals. Chem. Eur. J..

[36] McKinnon J.J., Jayatilaka D., Spackman M.A. (2007). Towards quantitative analysis of intermolecular interactions with Hirshfeld surfaces. Chem. Commun..

[37] Ureña F., Gómez M., González J., Torres E. (2003). A new insight into the vibrational analysis of pyridine. Acta Part A Mol. Biomol. Spectrosc..

[38] Wiberg K.B., Walters V.A., Wong K.N., Colson S.D. (1984). Azines: Vibrational force field and intensities for pyridine. J. Phys. Chem..

[39] Lorenc J., Bryndal I., Syska W., Wandas M., Marchewka M., Pietraszko A., Lis T., Mączka M., Hermanowicz K., Hanuza J. (2010). Order–disorder phase transitions and their influence on the structure and vibrational properties of new hybrid material: 2-Amino-4-methyl-3-nitropyridinium trifluoroacetate. Chem. Phys..

[40] Lorenc J., Hanuza J., Janczak J. (2012). Structural and vibrational study of 3- or 5-methyl substituted 2-*N*-ethylamino-4-nitropyridine N-oxides. Vib. Spectrosc..

[41] Bryndal I., Lorenc J., Macalik L., Michalski J., Sąsiadek W., Lis T., Hanuza J. (2019). Crystal structure, vibrational and optic properties of 2-*N*-methylamino-3-methylpyridine N-oxide–Its X-ray and spectroscopic studies as well as DFT quantum chemical calculations. J. Mol. Struct..

[42] Socrates G. (2004). Infrared and Raman Characteristic Group Frequencies: Tables and Charts.

[43] Bryndal I., Kucharska E., Sąsiadek W., Wandas M., Lis T., Lorenc J., Hanuza J. (2012). Molecular and crystal structures, vibrational studies and quantum chemical calculations of 3 and 5-nitroderivatives of 2-amino-4-methylpyridine. Spectrochim. Acta Part A Mol. Biomol. Spectrosc..

[44] Bryndal I., Marchewka M., Wandas M., Sąsiadek W., Lorenc J., Lis T., Dymińska L., Kucharska E., Hanuza J. (2014). The role of hydrogen bonds in the crystals of 2-amino-4-methyl-5-nitropyridinium trifluoroacetate monohydrate and 4-hydroxybenzenesulfonate–X-ray and spectroscopic studies. Spectrochim. Acta Part A Mol. Biomol. Spectrosc..

[45] Lorenc J., Kucharska E., Hanuza J., Chojnacki H. (2004). Excited electronic states of 2-ethylamino-(3 or 5)-methyl-4-nitropyridine and 2-methylamino-(3 or 5)-methyl-4-nitropyridine. J. Mol. Struct..

[46] Lorenc J., Zając A., Janczak J., Lisiecki R., Hanuza J., Hermanowicz K. (2022). Structure and optical properties of new nitro-derivatives of 2-*N*-alkiloamino-picoline N-oxide isomers. J. Mol. Struct..

[47] Barakat A., Al-Najjar H.J., Al-Majid A.M., Soliman S.M., Mabkhot Y.N., Shaik M.R., Ghabbour H.A., Fun H.-K. (2015). Synthesis, NMR, FT-IR, X-ray structural characterization, DFT analysis and isomerism aspects of 5-(2,6-dichlorobenzylidene)pyrimidine-2,4,6(1H,3H,5H)-trione. Spectrochim. Acta Part A Mol. Biomol. Spectrosc..

[48] Carthigayan K., Xavier S., Periandy S. (2015). HOMO–LUMO, UV, NLO, NMR and vibrational analysis of 3-methyl-1-phenylpyrazole using FT-IR, FT-RAMAN FT-NMR spectra and HF-DFT computational methods. Spectrochim. Acta Part A Mol. Biomol. Spectrosc..

[49] Puszko A., Wasylina L. (1995). The influence of steric effect on 1H NMR, 13C NMR and IR spectra of methylated de-rivatives of 4-nitropyridine N-oxide. Chem. Pap..

[50] Kątcka M., Urbański T. (1968). NMR spectra of pyridine, picolines and hydrochlorides and of their hydrochlorides and methiodides. Bull. Acad. Pol. Sci..

[51] Palasek B., Talik Z. (1985). Synthesis of the 2-alcilonitramino-5-nitroderivatives of 6-methylpyridine. Pr. Nauk. AE Wrocław.

[52] (2023). Rigaku Oxford Diffraction, CrysAlisPro 1.171.42.93a Software System, Oxford, UK. https://rigaku.com/products/crystallography/x-ray-diffraction/crysalispro.

[53] Sheldrick G.M. (2015). SHELXT–Integrated space-group and crystal-structure determination. Acta Cryst..

[54] Sheldrick G.M. (2015). Crystal Structure of Refinement with SHELXL. Acta Cryst..

[55] Brandenburg K., Putz H. (2006). DIAMOND.

[56] Wolf S.W., Grimwood D.J., MacKimon J.J., Turner M.J., Jayatilaka D., Spackman A.M. (2013). Crystal Explorer.

[57] Frisch M.J., Trucks G.W., Schlegel H.B., Scuseria G.E., Robb M.A., Cheeseman J.R., Montgomery J.A., Vreven T., Kudin K.N., Burant J.C. (2003). Gaussian 03, Revision A.1.

[58] Becke A.D. (1996). Density-functional thermochemistry. IV. A new dynamical correlation functional and implications for exact-exchange mixing. J. Chem. Phys..

[59] Lee C., Yang W., Parr R.G. (1988). Density-functional exchange-energy approximation with correct asymptotic behavior. Phys. Rev..

[60] Parr R.G., Yang W. (1989). Density-Functional Theory of Atoms and Molecules.

[61] McLean A.D., Chandler G.S. (1980). Contracted Gaussian basis sets for molecular calculations. I. Second row atoms, Z = 11–18. J. Chem. Phys..

[62] Krishnan R., Binkley J.S., Seeger R., Pople J.A. (1980). Self-consistent molecular orbital methods. XX. A basis set for correlated wave functions. J. Chem. Phys..

[63] Rostkowska H., Lapinski L., Nowak M.J. (2009). Analysis of the normal modes of molecules with D3h symmetry: Infrared spectra of monomeric s-triazine and cyanuric acid. Vib. Spectrosc..

[64] (2009). GaussView 4.1.

[65] Zhurko G.A., Zhurko D.A. Chemcraft Graphical Program for Visualization of Computed Results. http://www.chemcraftprog.com.

[66] Michalska D. (2002). RAINT (Raman Intensities), Computer Program for Calculation of Raman Intensities from the Gaussian Outputs.

[67] Palafox M.A., Rastogi V. (2002). Quantum chemical predictions of the vibrational spectra of polyatomic molecules. The uracil molecule and two derivatives. Spectrochim. Acta.

[68] Joshi K.K., Pal S. (2022). Chemistry with Schiff bases of pyridine derivatives: Their potential as bioactive ligands and chemosensors. The Edited Volume: Exploring Chemistry with Pyridine Derivatives.

