# Supplementary material for: Structure and Optical Properties of New 2-N-Phenylamino-methyl-nitro-pyridine Isomers
Source: Int J Mol Sci. 2025 Mar 21;26(7):2874. doi: 10.3390/ijms26072874 (PMC11989132; doi:10.3390/ijms26072874)

## Supplementary Materials

# Structure and optical properties of new 2-N-phenylamino-methyl-nitro-pyridine isomers

**Patrycja Godlewska [1,*], Jerzy Hanuza [2,*], Jan Janczak [2], Radosław Lisiecki [2], Małgorzata Basiak [1], Adam Zając [1], Lucyna. Dymińska [2],**

[1] Department of Bioorganic Chemistry, Faculty of Production Engineering, Wroclaw University of Economics and Business, 118-120 Komandorska str., 53-345 Wrocław, Poland

[2] Institute of Low Temperature and Structure Research, 2 Okólna str., 50-422 Wrocław, Poland

**Table S1**. Optimized parameters (Å,°) for 2-*N*-phenylamino-3-nitro-4-methylpyridine (PA3N4MP- **1**).

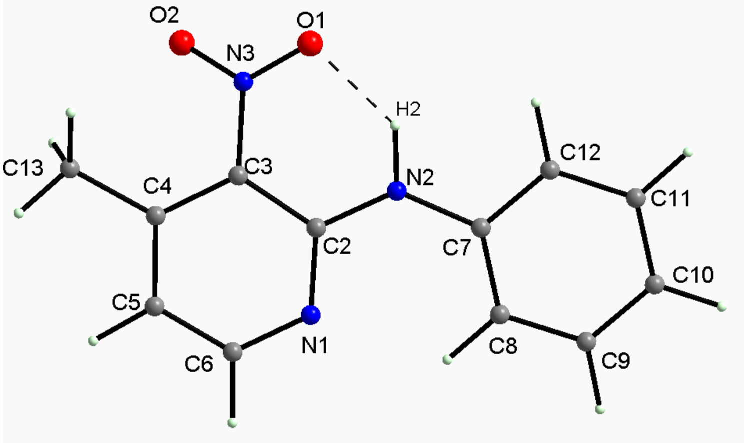


| | | | | | |
|---|---|---|---|---|---|
| N3–O1 | 1.242 | N3–O2 | 1.226 | N1–C2 | 1.343 |
| C2–C3 | 1.443 | C3–N3 | 1.454 | C3–C4 | 1.410 |
| C4–C13 | 1.508 | C4–C5 | 1.394 | C5–C6 | 1.390 |
| C6–N1 | 1.325 | C2–N2 | 1.360 | N2–C7 | 1.411 |
| C7–C8 | 1.401 | C8–C9 | 1.394 | C9–C10 | 1.392 |
| C10–C11 | 1.395 | C11–C12 | 1.388 | C12–C7 | 1.406 |
| C5–H5 | 1.082 | C6–H6 | 1.087 | C8–H8 | 1.077 |
| C9–H9 | 1.084 | C10–H10 | 1.084 | C11–H11 | 1.084 |
| C12–H12 | 1.085 | C13–H13A | 1.091 | C13–H13B | 1.091 |
| C13–H13C | 1.091 | | | | |
| O1–N3–O2 | 122.37 | O1–N3–C3 | 118.78 | N1–C2–C3 | 120.12 |
| C2–C3–C4 | 120.07 | C3–C4–C13 | 125.11 | C3–C4–C5 | 116.76 |
| C4–C5–C6 | 119.52 | C5–C6–N1 | 124.30 | C6–N1–C2 | 119.08 |
| N1–C2–N2 | 118.28 | C2–N2–H2 | 113.16 | C7–N2–H2 | 115.16 |
| C2–N2–C7 | 131.64 | N2–C7–C8 | 125.50 | C7–C8–C9 | 119.52 |
| C8–C9–C10 | 121.39 | C9–C10–C11 | 119.04 | C10–C11–C12 | 120.27 |
| C12–C7–N2 | 120.75 | | | | |
| N2–C2–C3–N3 | 5.17 | C2–C3–N3–O1 | -25.14 | C2–C3–N3–O2 | 154.28 |
| C2–C3–C4–C13 | 176.42 | C3–C4–C5–C6 | -1.43 | C4–C5–C6–N1 | 2.70 |
| C5–C6–N1–C2 | -0.21 | C6–N1–C2–N2 | 177.35 | N1–C2–N2–C7 | 5.59 |
| C2–N2–C7–C12 | -171.16 | C2–N2–C7–C8 | 10.44 | N2–C7–C8–C9 | 178.41 |
| C7–C8–C9–C10 | 0.01 | C8–C9–C10–C11 | -0.09 | C9–C10–C11–C12 | 0.08 |
| C10–C11–C12–C7 | 0.01 | | | | |

Intramolecular hydrogen bond

| | | | | | | | |
|---|---|---|---|---|---|---|---|
| N2–H2 | 1.014 | H2···O1 | 1.814 | N2···O1 | 2.632 | N2–H2···O1 | 135.17 |

Angle between the plane of $NO_2$ (O1N3O2)and the plane of pyridine ring(N1,C2-C6) 25.50

Angle between the plane of pyridine ring (N1,C2-C6) and the plane of phenyl ring (C7-C12) 15.70

Optimization completed.

```
                           ----------------------------
                           !   Optimized Parameters   !
                           ! (Angstroms and Degrees)  !
 --------------------------                            --------------------------
 ! Name  Definition              Value          Derivative Info.                !
 --------------------------------------------------------------------------------
 ! R1    R(1,2)                  1.3431         -DE/DX =    0.0                 !
 ! R2    R(1,7)                  1.3251         -DE/DX =    0.0                 !
 ! R3    R(2,3)                  1.4433         -DE/DX =    0.0                 !
 ! R4    R(2,9)                  1.3599         -DE/DX =    0.0                 !
 ! R5    R(3,4)                  1.41           -DE/DX =    0.0                 !
 ! R6    R(3,22)                 1.454          -DE/DX =    0.0                 !
 ! R7    R(4,5)                  1.3939         -DE/DX =    0.0                 !
 ! R8    R(4,25)                 1.5078         -DE/DX =    0.0                 !
 ! R9    R(5,6)                  1.0824         -DE/DX =    0.0                 !
 ! R10   R(5,7)                  1.3895         -DE/DX =    0.0                 !
 ! R11   R(7,8)                  1.0872         -DE/DX =    0.0                 !
 ! R12   R(9,10)                 1.0141         -DE/DX =    0.0                 !
 ! R13   R(9,11)                 1.4115         -DE/DX =    0.0                 !
 ! R14   R(11,12)                1.4013         -DE/DX =    0.0                 !
 ! R15   R(11,20)                1.4056         -DE/DX =    0.0                 !
 ! R16   R(12,13)                1.0772         -DE/DX =    0.0                 !
 ! R17   R(12,14)                1.394          -DE/DX =    0.0                 !
 ! R18   R(14,15)                1.0845         -DE/DX =    0.0                 !
 ! R19   R(14,16)                1.3922         -DE/DX =    0.0                 !
 ! R20   R(16,17)                1.0836         -DE/DX =    0.0                 !
 ! R21   R(16,18)                1.3949         -DE/DX =    0.0                 !
 ! R22   R(18,19)                1.0841         -DE/DX =    0.0                 !
 ! R23   R(18,20)                1.3882         -DE/DX =    0.0                 !
 ! R24   R(20,21)                1.0854         -DE/DX =    0.0                 !
 ! R25   R(22,23)                1.2421         -DE/DX =    0.0                 !
 ! R26   R(22,24)                1.2256         -DE/DX =    0.0                 !
 ! R27   R(25,26)                1.0906         -DE/DX =    0.0                 !
 ! R28   R(25,27)                1.0909         -DE/DX =    0.0                 !
 ! R29   R(25,28)                1.0913         -DE/DX =    0.0                 !
 ! A1    A(2,1,7)              119.0782         -DE/DX =    0.0                 !
 ! A2    A(1,2,3)              120.1217         -DE/DX =    0.0                 !
 ! A3    A(1,2,9)              118.2802         -DE/DX =    0.0                 !
 ! A4    A(3,2,9)              121.5947         -DE/DX =    0.0                 !
 ! A5    A(2,3,4)              120.0706         -DE/DX =    0.0                 !
 ! A6    A(2,3,22)             120.7344         -DE/DX =    0.0                 !
 ! A7    A(4,3,22)             119.18           -DE/DX =    0.0                 !
 ! A8    A(3,4,5)              116.7545         -DE/DX =    0.0                 !
 ! A9    A(3,4,25)             125.1081         -DE/DX =    0.0                 !
 ! A10   A(5,4,25)             118.1178         -DE/DX =    0.0                 !
 ! A11   A(4,5,6)              120.1629         -DE/DX =    0.0                 !
 ! A12   A(4,5,7)              119.5174         -DE/DX =    0.0                 !
 ! A13   A(6,5,7)              120.3193         -DE/DX =    0.0                 !
 ! A14   A(1,7,5)              124.2995         -DE/DX =    0.0                 !
 ! A15   A(1,7,8)              115.645          -DE/DX =    0.0                 !
 ! A16   A(5,7,8)              120.0493         -DE/DX =    0.0                 !
 ! A17   A(2,9,10)             113.1558         -DE/DX =    0.0                 !
 ! A18   A(2,9,11)             131.6388         -DE/DX =    0.0                 !
 ! A19   A(10,9,11)            115.1574         -DE/DX =    0.0                 !
 ! A20   A(9,11,12)            125.4979         -DE/DX =    0.0                 !
 ! A21   A(9,11,20)            115.4508         -DE/DX =    0.0                 !
 ! A22   A(12,11,20)           119.0324         -DE/DX =    0.0                 !
 ! A23   A(11,12,13)           119.8023         -DE/DX =    0.0                 !
 ! A24   A(11,12,14)           119.5209         -DE/DX =    0.0                 !
```

```
! A25   A(13,12,14)          120.6735         -DE/DX =    0.0                 !
! A26   A(12,14,15)          118.7241         -DE/DX =    0.0                 !
! A27   A(12,14,16)          121.393          -DE/DX =    0.0                 !
! A28   A(15,14,16)          119.8829         -DE/DX =    0.0                 !
! A29   A(14,16,17)          120.5271         -DE/DX =    0.0                 !
! A30   A(14,16,18)          119.0376         -DE/DX =    0.0                 !
! A31   A(17,16,18)          120.4353         -DE/DX =    0.0                 !
! A32   A(16,18,19)          120.3333         -DE/DX =    0.0                 !
! A33   A(16,18,20)          120.2678         -DE/DX =    0.0                 !
! A34   A(19,18,20)          119.3989         -DE/DX =    0.0                 !
! A35   A(11,20,18)          120.7482         -DE/DX =    0.0                 !
! A36   A(11,20,21)          119.4476         -DE/DX =    0.0                 !
! A37   A(18,20,21)          119.8042         -DE/DX =    0.0                 !
! A38   A(3,22,23)           118.7806         -DE/DX =    0.0                 !
! A39   A(3,22,24)           118.8503         -DE/DX =    0.0                 !
! A40   A(23,22,24)          122.3665         -DE/DX =    0.0                 !
! A41   A(4,25,26)           112.2736         -DE/DX =    0.0                 !
! A42   A(4,25,27)           109.1538         -DE/DX =    0.0                 !
! A43   A(4,25,28)           111.0073         -DE/DX =    0.0                 !
! A44   A(26,25,27)          108.9673         -DE/DX =    0.0                 !
! A45   A(26,25,28)          106.4724         -DE/DX =    0.0                 !
! A46   A(27,25,28)          108.8769         -DE/DX =    0.0                 !
! D1    D(7,1,2,3)            -3.308          -DE/DX =    0.0                 !
! D2    D(7,1,2,9)           177.3526         -DE/DX =    0.0                 !
! D3    D(2,1,7,5)            -0.2091         -DE/DX =    0.0                 !
! D4    D(2,1,7,8)          -179.2963         -DE/DX =    0.0                 !
! D5    D(1,2,3,4)             4.4355         -DE/DX =    0.0                 !
! D6    D(1,2,3,22)         -174.1435         -DE/DX =    0.0                 !
! D7    D(9,2,3,4)          -176.2474         -DE/DX =    0.0                 !
! D8    D(9,2,3,22)            5.1736         -DE/DX =    0.0                 !
! D9    D(1,2,9,10)         -171.7174         -DE/DX =    0.0                 !
! D10   D(1,2,9,11)            5.5916         -DE/DX =    0.0                 !
! D11   D(3,2,9,10)            8.9534         -DE/DX =    0.0                 !
! D12   D(3,2,9,11)         -173.7376         -DE/DX =    0.0                 !
! D13   D(2,3,4,5)            -1.9342         -DE/DX =    0.0                 !
! D14   D(2,3,4,25)          176.4201         -DE/DX =    0.0                 !
! D15   D(22,3,4,5)          176.6669         -DE/DX =    0.0                 !
! D16   D(22,3,4,25)          -4.9788         -DE/DX =    0.0                 !
! D17   D(2,3,22,23)         -25.1423         -DE/DX =    0.0                 !
! D18   D(2,3,22,24)         154.2841         -DE/DX =    0.0                 !
! D19   D(4,3,22,23)         156.2662         -DE/DX =    0.0                 !
! D20   D(4,3,22,24)         -24.3074         -DE/DX =    0.0                 !
! D21   D(3,4,5,6)           178.8134         -DE/DX =    0.0                 !
! D22   D(3,4,5,7)            -1.4303         -DE/DX =    0.0                 !
! D23   D(25,4,5,6)            0.3398         -DE/DX =    0.0                 !
! D24   D(25,4,5,7)         -179.9039         -DE/DX =    0.0                 !
! D25   D(3,4,25,26)         -43.4482         -DE/DX =    0.0                 !
! D26   D(3,4,25,27)        -164.3845         -DE/DX =    0.0                 !
! D27   D(3,4,25,28)          75.5983         -DE/DX =    0.0                 !
! D28   D(5,4,25,26)         134.8856         -DE/DX =    0.0                 !
! D29   D(5,4,25,27)          13.9494         -DE/DX =    0.0                 !
! D30   D(5,4,25,28)        -106.0678         -DE/DX =    0.0                 !
! D31   D(4,5,7,1)             2.6968         -DE/DX =    0.0                 !
! D32   D(4,5,7,8)          -178.2539         -DE/DX =    0.0                 !
! D33   D(6,5,7,1)          -177.5473         -DE/DX =    0.0                 !
! D34   D(6,5,7,8)             1.502          -DE/DX =    0.0                 !
! D35   D(2,9,11,12)          10.4439         -DE/DX =    0.0                 !
! D36   D(2,9,11,20)        -171.1634         -DE/DX =    0.0                 !
! D37   D(10,9,11,12)       -172.2896         -DE/DX =    0.0                 !
```

```
! D38   D(10,9,11,20)           6.1031         -DE/DX =    0.0                 !
! D39   D(9,11,12,13)          -2.239          -DE/DX =    0.0                 !
! D40   D(9,11,12,14)         178.4122         -DE/DX =    0.0                 !
! D41   D(20,11,12,13)        179.4209         -DE/DX =    0.0                 !
! D42   D(20,11,12,14)          0.0721         -DE/DX =    0.0                 !
! D43   D(9,11,20,18)        -178.583          -DE/DX =    0.0                 !
! D44   D(9,11,20,21)           1.3493         -DE/DX =    0.0                 !
! D45   D(12,11,20,18)         -0.0796         -DE/DX =    0.0                 !
! D46   D(12,11,20,21)        179.8527         -DE/DX =    0.0                 !
! D47   D(11,12,14,15)        179.9762         -DE/DX =    0.0                 !
! D48   D(11,12,14,16)          0.0134         -DE/DX =    0.0                 !
! D49   D(13,12,14,15)          0.6332         -DE/DX =    0.0                 !
! D50   D(13,12,14,16)       -179.3296         -DE/DX =    0.0                 !
! D51   D(12,14,16,17)        179.9199         -DE/DX =    0.0                 !
! D52   D(12,14,16,18)         -0.0921         -DE/DX =    0.0                 !
! D53   D(15,14,16,17)         -0.0424         -DE/DX =    0.0                 !
! D54   D(15,14,16,18)        179.9455         -DE/DX =    0.0                 !
! D55   D(14,16,18,19)       -179.9205         -DE/DX =    0.0                 !
! D56   D(14,16,18,20)          0.0845         -DE/DX =    0.0                 !
! D57   D(17,16,18,19)          0.0674         -DE/DX =    0.0                 !
! D58   D(17,16,18,20)       -179.9276         -DE/DX =    0.0                 !
! D59   D(16,18,20,11)          0.0008         -DE/DX =    0.0                 !
! D60   D(16,18,20,21)       -179.9313         -DE/DX =    0.0                 !
! D61   D(19,18,20,11)       -179.9943         -DE/DX =    0.0                 !
! D62   D(19,18,20,21)          0.0736         -DE/DX =    0.0                 !
--------------------------------------------------------------------------------
```

Optimized Parameters (xyz)

| Center Number | Atomic Number | Atomic Type | Coordinates (Angstroms) X | Y | Z |
|---|---|---|---|---|---|
| 1 | 7 | 0 | -0.030221 | -0.020182 | -0.040740 |
| 2 | 6 | 0 | 1.307905 | -0.115503 | 0.025014 |
| 3 | 6 | 0 | 1.938112 | -1.410661 | 0.117796 |
| 4 | 6 | 0 | 1.157832 | -2.582822 | 0.045574 |
| 5 | 6 | 0 | -0.220440 | -2.412747 | -0.074133 |
| 6 | 1 | 0 | -0.869997 | -3.274990 | -0.153311 |
| 7 | 6 | 0 | -0.753428 | -1.129574 | -0.085855 |
| 8 | 1 | 0 | -1.829340 | -0.987560 | -0.150237 |
| 9 | 7 | 0 | 2.036882 | 1.032505 | 0.015347 |
| 10 | 1 | 0 | 3.034606 | 0.870658 | -0.066742 |
| 11 | 6 | 0 | 1.649899 | 2.389463 | 0.048902 |
| 12 | 6 | 0 | 0.356052 | 2.860417 | 0.309279 |
| 13 | 1 | 0 | -0.446477 | 2.159989 | 0.469585 |
| 14 | 6 | 0 | 0.119059 | 4.233711 | 0.344462 |
| 15 | 1 | 0 | -0.887173 | 4.583940 | 0.546711 |
| 16 | 6 | 0 | 1.142268 | 5.152032 | 0.125718 |
| 17 | 1 | 0 | 0.942008 | 6.216588 | 0.155053 |
| 18 | 6 | 0 | 2.430136 | 4.682142 | -0.131733 |
| 19 | 1 | 0 | 3.242859 | 5.378656 | -0.303792 |
| 20 | 6 | 0 | 2.682471 | 3.317631 | -0.169992 |
| 21 | 1 | 0 | 3.687706 | 2.960276 | -0.369881 |
| 22 | 7 | 0 | 3.373801 | -1.525903 | 0.316797 |
| 23 | 8 | 0 | 4.105586 | -0.592906 | -0.053031 |
| 24 | 8 | 0 | 3.817431 | -2.534856 | 0.852755 |
| 25 | 6 | 0 | 1.702360 | -3.988811 | 0.052611 |
| 26 | 1 | 0 | 2.577155 | -4.096528 | -0.589719 |
| 27 | 1 | 0 | 0.925781 | -4.675112 | -0.287900 |
| 28 | 1 | 0 | 2.017108 | -4.281444 | 1.055771 |

**Table S2**. Optimized parameters (Å,°) for 2-N-phenylamino-3-nitro-6-methylpyridine, $C_{12}H_{11}N_3O_2$ (PA3N6MP - **2**)

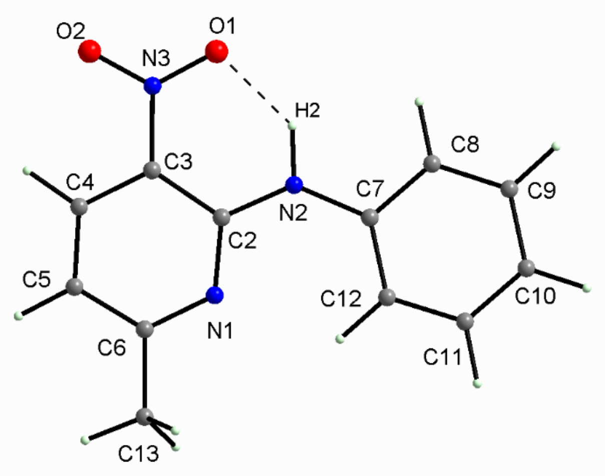


| | | | | | |
|---|---|---|---|---|---|
| N3–O1 | 1.243 | N3–O2 | 1.226 | N1–C2 | 1.344 |
| C2–C3 | 1.438 | C3–N3 | 1.450 | C3–C4 | 1.393 |
| C4–C5 | 1.380 | C5–C6 | 1.404 | C6–N1 | 1.331 |
| C6–C13 | 1.505 | C2–N2 | 1.358 | N2–C7 | 1.410 |
| C7–C8 | 1.406 | C8–C9 | 1.388 | C9–C10 | 1.395 |
| C10–C11 | 1.392 | C11–C12 | 1.394 | C12–C7 | 1.401 |
| C4–H4 | 1.081 | C5–H5 | 1.082 | C8–H8 | 1.085 |
| C9–H9 | 1.084 | C10–H10 | 1.084 | C11–H11 | 1.084 |
| C12–H12 | 1.077 | C13–H13A | 1.093 | C13–H13B | 1.091 |
| C13–H13C | 1.093 | | | | |

| | | | | | |
|---|---|---|---|---|---|
| O1–N3–O2 | 122.61 | O1–N3–C3 | 118.69 | N1–C2–C3 | 119.38 |
| C2–C3–C4 | 118.92 | C3–C4–C5 | 120.01 | C4–C5–C6 | 118.13 |
| C5–C6–C13 | 121.31 | C5–C6–N1 | 122.47 | C13–C6–N1 | 116.21 |
| N1–C2–N2 | 119.23 | C2–N2–C7 | 132.24 | C2–N2–H2 | 112.41 |
| N2–C7–C8 | 115.37 | C7–C8–C9 | 120.74 | C8–C9–C10 | 120.29 |
| C9–C10–C11 | 119.03 | C10–C11–C12 | 121.41 | C11–C12–C7 | 119.52 |
| C12–C7–C8 | 119.02 | C12–C7–N2 | 125.59 | | |

| | | | | | |
|---|---|---|---|---|---|
| N2–C2–C3–N3 | 0.01 | C2–C3–N3–O1 | 0.01 | C2–C3–N3–O2 | -179.99 |
| C2–C3–C4–C5 | 0.00 | C3–C4–C5–C6 | -0.01 | C4–C5–C6–N1 | 0.01 |
| C5–C6–N1–C2 | 0.00 | C6–N1–C2–N2 | 179.99 | N1–C2–N2–C7 | 0.01 |
| C2–N2–C7–C8 | 179.99 | N2–C7–C8–C9 | 179.99 | C7–C8–C9–C10 | 0.00 |
| C8–C9–C10–C11 | 0.00 | C9–C10–C11–C12 | 0.00 | C10–C11–C12–C7 | 0.00 |

intramolecular hydrogen bond

| | | | | | | | |
|---|---|---|---|---|---|---|---|
| N2–H2 | 1.016 | H2···O1 | 1.818 | N2···O1 | 2.649 | N2–H2···O1 | 136.58 |

The conformation of the whole molecule is planar (without H atoms of $CH_3$).

```
Optimization completed.

                              ----------------------------
                              !   Optimized Parameters   !
                              ! (Angstroms and Degrees)  !
 --------------------------                            --------------------------
 ! Name  Definition              Value          Derivative Info.                !
 --------------------------------------------------------------------------------
 ! R1    R(1,2)                  1.3439         -DE/DX =    0.0                 !
 ! R2    R(1,8)                  1.3314         -DE/DX =    0.0                 !
```

```
! R3    R(2,3)                  1.4376         -DE/DX =    0.0                 !
! R4    R(2,9)                  1.3578         -DE/DX =    0.0                 !
! R5    R(3,4)                  1.3931         -DE/DX =    0.0                 !
! R6    R(3,22)                 1.4502         -DE/DX =    0.0                 !
! R7    R(4,5)                  1.0814         -DE/DX =    0.0                 !
! R8    R(4,6)                  1.3804         -DE/DX =    0.0                 !
! R9    R(6,7)                  1.0819         -DE/DX =    0.0                 !
! R10   R(6,8)                  1.4035         -DE/DX =    0.0                 !
! R11   R(8,25)                 1.5043         -DE/DX =    0.0                 !
! R12   R(9,10)                 1.0159         -DE/DX =    0.0                 !
! R13   R(9,11)                 1.4105         -DE/DX =    0.0                 !
! R14   R(11,12)                1.406          -DE/DX =    0.0                 !
! R15   R(11,20)                1.4014         -DE/DX =    0.0                 !
! R16   R(12,13)                1.0854         -DE/DX =    0.0                 !
! R17   R(12,14)                1.3879         -DE/DX =    0.0                 !
! R18   R(14,15)                1.0841         -DE/DX =    0.0                 !
! R19   R(14,16)                1.3951         -DE/DX =    0.0                 !
! R20   R(16,17)                1.0836         -DE/DX =    0.0                 !
! R21   R(16,18)                1.3919         -DE/DX =    0.0                 !
! R22   R(18,19)                1.0845         -DE/DX =    0.0                 !
! R23   R(18,20)                1.3943         -DE/DX =    0.0                 !
! R24   R(20,21)                1.077          -DE/DX =    0.0                 !
! R25   R(22,23)                1.2261         -DE/DX =    0.0                 !
! R26   R(22,24)                1.2433         -DE/DX =    0.0                 !
! R27   R(25,26)                1.0933         -DE/DX =    0.0                 !
! R28   R(25,27)                1.0906         -DE/DX =    0.0                 !
! R29   R(25,28)                1.0933         -DE/DX =    0.0                 !
! A1    A(2,1,8)              121.0978         -DE/DX =    0.0                 !
! A2    A(1,2,3)              119.3757         -DE/DX =    0.0                 !
! A3    A(1,2,9)              119.2257         -DE/DX =    0.0                 !
! A4    A(3,2,9)              121.3987         -DE/DX =    0.0                 !
! A5    A(2,3,4)              118.9156         -DE/DX =    0.0                 !
! A6    A(2,3,22)             124.0585         -DE/DX =    0.0                 !
! A7    A(4,3,22)             117.0259         -DE/DX =    0.0                 !
! A8    A(3,4,5)              118.242          -DE/DX =    0.0                 !
! A9    A(3,4,6)              120.0067         -DE/DX =    0.0                 !
! A10   A(5,4,6)              121.7513         -DE/DX =    0.0                 !
! A11   A(4,6,7)              120.997          -DE/DX =    0.0                 !
! A12   A(4,6,8)              118.1288         -DE/DX =    0.0                 !
! A13   A(7,6,8)              120.8741         -DE/DX =    0.0                 !
! A14   A(1,8,6)              122.4755         -DE/DX =    0.0                 !
! A15   A(1,8,25)             116.2121         -DE/DX =    0.0                 !
! A16   A(6,8,25)             121.3124         -DE/DX =    0.0                 !
! A17   A(2,9,10)             112.4105         -DE/DX =    0.0                 !
! A18   A(2,9,11)             132.2435         -DE/DX =    0.0                 !
! A19   A(10,9,11)            115.346          -DE/DX =    0.0                 !
! A20   A(9,11,12)            115.3899         -DE/DX =    0.0                 !
! A21   A(9,11,20)            125.5889         -DE/DX =    0.0                 !
! A22   A(12,11,20)           119.0212         -DE/DX =    0.0                 !
! A23   A(11,12,13)           119.4661         -DE/DX =    0.0                 !
! A24   A(11,12,14)           120.7377         -DE/DX =    0.0                 !
! A25   A(13,12,14)           119.7963         -DE/DX =    0.0                 !
! A26   A(12,14,15)           119.3801         -DE/DX =    0.0                 !
! A27   A(12,14,16)           120.2935         -DE/DX =    0.0                 !
! A28   A(15,14,16)           120.3264         -DE/DX =    0.0                 !
! A29   A(14,16,17)           120.4241         -DE/DX =    0.0                 !
! A30   A(14,16,18)           119.0261         -DE/DX =    0.0                 !
! A31   A(17,16,18)           120.5498         -DE/DX =    0.0                 !
! A32   A(16,18,19)           119.8762         -DE/DX =    0.0                 !
```

```
! A33   A(16,18,20)          121.4012         -DE/DX =    0.0                 !
! A34   A(19,18,20)          118.7227         -DE/DX =    0.0                 !
! A35   A(11,20,18)          119.5204         -DE/DX =    0.0                 !
! A36   A(11,20,21)          119.6292         -DE/DX =    0.0                 !
! A37   A(18,20,21)          120.8504         -DE/DX =    0.0                 !
! A38   A(3,22,23)           118.7011         -DE/DX =    0.0                 !
! A39   A(3,22,24)           118.6853         -DE/DX =    0.0                 !
! A40   A(23,22,24)          122.6136         -DE/DX =    0.0                 !
! A41   A(8,25,26)           110.0258         -DE/DX =    0.0                 !
! A42   A(8,25,27)           111.7814         -DE/DX =    0.0                 !
! A43   A(8,25,28)           110.0281         -DE/DX =    0.0                 !
! A44   A(26,25,27)          108.858          -DE/DX =    0.0                 !
! A45   A(26,25,28)          107.1655         -DE/DX =    0.0                 !
! A46   A(27,25,28)          108.8587         -DE/DX =    0.0                 !
! D1    D(8,1,2,3)            -0.0027         -DE/DX =    0.0                 !
! D2    D(8,1,2,9)           179.9952         -DE/DX =    0.0                 !
! D3    D(2,1,8,6)            -0.0017         -DE/DX =    0.0                 !
! D4    D(2,1,8,25)          179.9972         -DE/DX =    0.0                 !
! D5    D(1,2,3,4)             0.0046         -DE/DX =    0.0                 !
! D6    D(1,2,3,22)         -179.9941         -DE/DX =    0.0                 !
! D7    D(9,2,3,4)          -179.9933         -DE/DX =    0.0                 !
! D8    D(9,2,3,22)            0.008          -DE/DX =    0.0                 !
! D9    D(1,2,9,10)          179.9926         -DE/DX =    0.0                 !
! D10   D(1,2,9,11)            0.0024         -DE/DX =    0.0                 !
! D11   D(3,2,9,10)           -0.0095         -DE/DX =    0.0                 !
! D12   D(3,2,9,11)          180.0003         -DE/DX =    0.0                 !
! D13   D(2,3,4,5)           179.9982         -DE/DX =    0.0                 !
! D14   D(2,3,4,6)            -0.0021         -DE/DX =    0.0                 !
! D15   D(22,3,4,5)           -0.003          -DE/DX =    0.0                 !
! D16   D(22,3,4,6)          179.9967         -DE/DX =    0.0                 !
! D17   D(2,3,22,23)         180.0035         -DE/DX =    0.0                 !
! D18   D(2,3,22,24)           0.0038         -DE/DX =    0.0                 !
! D19   D(4,3,22,23)           0.0047         -DE/DX =    0.0                 !
! D20   D(4,3,22,24)        -179.9949         -DE/DX =    0.0                 !
! D21   D(3,4,6,7)           179.999          -DE/DX =    0.0                 !
! D22   D(3,4,6,8)            -0.0021         -DE/DX =    0.0                 !
! D23   D(5,4,6,7)            -0.0013         -DE/DX =    0.0                 !
! D24   D(5,4,6,8)           179.9975         -DE/DX =    0.0                 !
! D25   D(4,6,8,1)             0.0042         -DE/DX =    0.0                 !
! D26   D(4,6,8,25)         -179.9946         -DE/DX =    0.0                 !
! D27   D(7,6,8,1)          -179.9969         -DE/DX =    0.0                 !
! D28   D(7,6,8,25)            0.0042         -DE/DX =    0.0                 !
! D29   D(1,8,25,26)         -58.9406         -DE/DX =    0.0                 !
! D30   D(1,8,25,27)        -180.0128         -DE/DX =    0.0                 !
! D31   D(1,8,25,28)          58.9125         -DE/DX =    0.0                 !
! D32   D(6,8,25,26)         121.0583         -DE/DX =    0.0                 !
! D33   D(6,8,25,27)          -0.0139         -DE/DX =    0.0                 !
! D34   D(6,8,25,28)        -121.0885         -DE/DX =    0.0                 !
! D35   D(2,9,11,12)         179.9994         -DE/DX =    0.0                 !
! D36   D(2,9,11,20)          -0.0012         -DE/DX =    0.0                 !
! D37   D(10,9,11,12)          0.0093         -DE/DX =    0.0                 !
! D38   D(10,9,11,20)       -179.9913         -DE/DX =    0.0                 !
! D39   D(9,11,12,13)         -0.001          -DE/DX =    0.0                 !
! D40   D(9,11,12,14)        179.9992         -DE/DX =    0.0                 !
! D41   D(20,11,12,13)       179.9996         -DE/DX =    0.0                 !
! D42   D(20,11,12,14)        -0.0002         -DE/DX =    0.0                 !
! D43   D(9,11,20,18)       -179.999          -DE/DX =    0.0                 !
! D44   D(9,11,20,21)          0.0015         -DE/DX =    0.0                 !
! D45   D(12,11,20,18)         0.0004         -DE/DX =    0.0                 !
```

```
! D46   D(12,11,20,21)       -179.9991         -DE/DX =    0.0                 !
! D47   D(11,12,14,15)        180.0            -DE/DX =    0.0                 !
! D48   D(11,12,14,16)         -0.0003         -DE/DX =    0.0                 !
! D49   D(13,12,14,15)          0.0002         -DE/DX =    0.0                 !
! D50   D(13,12,14,16)       -180.0001         -DE/DX =    0.0                 !
! D51   D(12,14,16,17)       -179.9994         -DE/DX =    0.0                 !
! D52   D(12,14,16,18)          0.0005         -DE/DX =    0.0                 !
! D53   D(15,14,16,17)          0.0004         -DE/DX =    0.0                 !
! D54   D(15,14,16,18)       -179.9997         -DE/DX =    0.0                 !
! D55   D(14,16,18,19)        179.9995         -DE/DX =    0.0                 !
! D56   D(14,16,18,20)         -0.0004         -DE/DX =    0.0                 !
! D57   D(17,16,18,19)         -0.0006         -DE/DX =    0.0                 !
! D58   D(17,16,18,20)        179.9996         -DE/DX =    0.0                 !
! D59   D(16,18,20,11)         -0.0001         -DE/DX =    0.0                 !
! D60   D(16,18,20,21)        179.9994         -DE/DX =    0.0                 !
! D61   D(19,18,20,11)       -180.0            -DE/DX =    0.0                 !
! D62   D(19,18,20,21)         -0.0004         -DE/DX =    0.0                 !
--------------------------------------------------------------------------------
```

Optimized Parameters (xyz)

| Center Number | Atomic Number | Atomic Type | Coordinates (Angstroms) X | Y | Z |
|---|---|---|---|---|---|
| 1 | 7 | 0 | -0.000392 | -0.020716 | -0.016252 |
| 2 | 6 | 0 | -1.322494 | 0.220072 | -0.014598 |
| 3 | 6 | 0 | -2.240726 | -0.886028 | -0.018637 |
| 4 | 6 | 0 | -1.732702 | -2.183223 | -0.024082 |
| 5 | 1 | 0 | -2.433194 | -3.007131 | -0.027053 |
| 6 | 6 | 0 | -0.367874 | -2.390114 | -0.025568 |
| 7 | 1 | 0 | 0.044000 | -3.390522 | -0.029760 |
| 8 | 6 | 0 | 0.471867 | -1.265520 | -0.021509 |
| 9 | 7 | 0 | -1.762392 | 1.504630 | -0.009099 |
| 10 | 1 | 0 | -2.776372 | 1.566766 | -0.008455 |
| 11 | 6 | 0 | -1.081752 | 2.740000 | -0.004408 |
| 12 | 6 | 0 | -1.903357 | 3.880965 | 0.000695 |
| 13 | 1 | 0 | -2.982191 | 3.762028 | 0.000766 |
| 14 | 6 | 0 | -1.349819 | 5.153695 | 0.005623 |
| 15 | 1 | 0 | -2.003969 | 6.018164 | 0.009506 |
| 16 | 6 | 0 | 0.035497 | 5.318601 | 0.005583 |
| 17 | 1 | 0 | 0.469934 | 6.311296 | 0.009435 |
| 18 | 6 | 0 | 0.849979 | 4.189906 | 0.000527 |
| 19 | 1 | 0 | 1.928655 | 4.302107 | 0.000421 |
| 20 | 6 | 0 | 0.310004 | 2.904383 | -0.004464 |
| 21 | 1 | 0 | 0.948566 | 2.037165 | -0.008364 |
| 22 | 7 | 0 | -3.683945 | -0.743527 | -0.017446 |
| 23 | 8 | 0 | -4.375623 | -1.755955 | -0.021141 |
| 24 | 8 | 0 | -4.170646 | 0.400495 | -0.012725 |
| 25 | 6 | 0 | 1.969388 | -1.408031 | -0.022924 |
| 26 | 1 | 0 | 2.391817 | -0.912393 | -0.901129 |
| 27 | 1 | 0 | 2.276294 | -2.454503 | -0.027066 |
| 28 | 1 | 0 | 2.392887 | -0.919032 | 0.858476 |

**Table S3.** Experimental and calculated wavenumbers with assignment of the observed bands to respective normal modes for monomer.

| PA3N4MP | | | | PA3N6MP | | | | |
|---|---|---|---|---|---|---|---|---|
| Exp. | | Calc. | | Exp. | | Exp. | | |
| IR | RS | IR | RS | IR | RS | IR | RS | Assignment |
| 3367 | 3367 | 3433m | | 3344 | 3344 | 3311w | | $\nu$N-H····O |
| | | 3304m | | | | | | |
| 3132 | 3132 | 3135m | | 3135 | 3135 | 3146vw | | $\nu(C–H)_{\phi}$ |
| | | | | 3100 | 3100 | | 3082w | |
| 3074 | 3074 | | | 3077 | 3077 | | | $\nu(C–H)_{\theta}$ |
| 3069 | 3069 | | | 3069 | 3069 | | 3067w | $\nu(C–H)_{\phi}$ |
| 3056 | 3056 | 3053w | 3056w | 3056 | 3056 | | | |
| 3046 | 3046 | | | 3046 | 3046 | 3047vw | | |
| 3038 | 3038 | 3036w | | 3038 | 3038 | | | |
| 3025 | 3025 | | | | | | | $\nu(C_{\tau}–H)_{\theta}$ |
| 3002 | 3002 | | | 3003 | 3003 | | | $\nu_{as}(CH_3)$ |
| 2994 | 2994 | 2988w | 2989w | | | | | |
| | | 2981w | | 2974 | 2974 | | | |
| 2937 | 2937 | 2932w | 2935w | 2924 | 2924 | 2914vw | 2915w | $\nu_s(CH_3)$ |
| | | | | | | 1619s | | |
| | | 1607s | 1607m | | | 1613s | 1606m | |
| | | | 1596m | | | 1596vs | 1591w | |
| 1585 | 1585 | 1579s | 1579w | 1589 | 1589 | 1580vs | 1585w | $\delta(CNH)_{\phi}+\nu(\phi)$ |
| 1574 | 1574 | 1566s | 1567w | 1578 | 1578 | | 1578w | $\delta(CNH)_{\theta}+\nu(\phi)+\nu_{as}(NO_2)$ |
| | | | | 1562 | 1562 | | | |
| 1549 | 1549 | 1548s | 1547m | 1548 | 1548 | 1548vw | 1545w | $\nu_{as}(NO_2)+\nu(\phi)+\nu(\theta)+\delta(C_{\phi}NC_{\theta})$ |
| 1543 | 1543 | | | | | | | |
| 1521 | 1521 | 1511vs | 1500w | 1519 | 1519 | 1502s | 1500w | |
| 1481 | 1481 | 1485w | | | | 1491m | 1486w | $\nu_{as}(NO_2)+\nu(\phi)+\nu(\theta)$ |
| 1471 | 1471 | | | 1472 | 1472 | | | $\nu_{as}(NO_2)+\nu(\theta)$ |
| | | 1448m | 1450w | 1466 | 1466 | 1465m | 1458w | |
| | | | | 1433 | 1433 | 1432w | 1434w | $\nu_{as}(NO_2)+\delta_{as}(CH_3)$ |
| 1436 | 1436 | 1437m | | | | | | |
| 1429 | 1429 | 1429sh | | 1430 | 1430 | | | $\delta_{as}(CH_3)+\delta(\theta)$ |
| 1423 | 1423 | | | 1423 | 1423 | | | |
| 1416 | 1416 | | | | | | | $\nu_{as}(NO_2)$ |
| | | | | 1403 | 1403 | 1396w | 1394v | $\nu_{as}(NO_2)+\delta_{as}(CH_3)+\delta(\phi)+\delta($ |

| | | | | | | | | |
|---|---|---|---|---|---|---|---|---|
| | | | | | | | w | $\theta$) |
| 1366 | 1366 | 1378 | 1378m | 1378 | 1378 | 1373w | 1371w | $\nu_s(NO_2)$ $\delta_s(CH_3)$ |
| 1350 | 1350 | 1361 | | 1357 | 1357 | 1357w | | $\nu(CN)_\phi+\nu_s(NO_2)+\delta(\phi)+\delta_s(CH_3)$ |
| | | 1341 | 1348w | | | | 1339w | |
| 1324 | 1324 | 1326 | 1326vw | 1309 | | 1329vw | 1315w | $\nu_s(NO_2)+\delta(\phi)+\delta(\theta)_+\delta_s(CH_3)$ |
| 1304 | 1304 | 1307vw | 1308w | 1304 | 1309 | 1314w | | |
| | | 1301vw | | | 1304 | 1302w | 1302w | |
| 1279 | 1279 | 1286vw | 1272vw | 1280 | 1280 | 1271w | 1271w | $\nu_s(NO_2)+\delta(CH)+\nu(\phi+\theta)+\delta_s(CH_3)$ |
| 1256 | 1256 | 1248sh | 1238vs | 1240 | 1240 | 1239vs | 1236vs | $\nu_s(NO_2)+\delta(CH)+\nu(\phi+\theta)+\delta_s(CH_3)$ |
| 1226 | 1226 | 1237m | 1212s | 1227 | 1227 | 1219vs | 1216s | $\nu(\phi)+\nu_s(NO)+\nu(CN)_{\phi\alpha}+\nu(CNH)_\phi$ |
| 1197 | 1197 | 1220s | 1192sh | 1195 | 1195 | 1186s | 1188s | $\nu_s(NO_2)+(CNH)_\theta$ |
| 1184 | 1184 | 1203sh | | | | | | $\nu_s(NO_2)+\nu(NCN)_\phi$ |
| | | 1177m | | | | 1177s | | |
| 1159 | 1159 | 1166sh | 1162w | 1159 | 1159 | 1159m | 1156m | $\nu_s(NO_2)+\delta(CH)_\phi$ |
| | | | | 1157 | 1157 | | | |
| 1137 | 1137 | 1143w | 1150vw | 1138 | 1138 | | | |
| | | | | 1136 | 1136 | | | |
| 1122 | 1122 | 1081vw | | | | 1110vw | 1084vw | $\delta(CH)_\theta$ |
| 1069 | 1069 | 1063m | 1062m | 1070 | 1070 | 1073w | 1073w | $\delta(CH)_\phi$ |
| 1044 | 1044 | 1048w | | 1051 | 1051 | | | $\delta(\phi)_\phi+\nu(NO_2)$ |
| 1027 | 1027 | 1035w | 1031vw | | | 1031w | 1034vw | |
| 1017 | 1017 | 1027w | | 1016 | 1016 | | | $\rho(CH_3)$ |
| | | | | 1015 | 1015 | | | |
| 1008 | 1008 | | 1001m | 1009 | 1009 | | | $\delta(\phi)_\theta+\rho(CH_3)$ |
| 977 | 977 | 998vw | | 977 | 977 | 997w | 998m | |
| | | 981w | 984w | | | | | |
| 964 | 964 | 964vw | | 963 | 963 | 963w | 964w | $\rho(CH)$ |
| 960 | 960 | | | 962 | 962 | | | $\rho(CH_3)$ |
| 957 | 957 | | | | | | | |
| 946 | 946 | | | 945 | 945 | | | $\delta(CH)_\theta$ |
| | | | | 944 | 944 | | | |
| 883 | 883 | 903w | | 883 | 883 | 903w | | |

| | | | | | | | | |
|---|---|---|---|---|---|---|---|---|
| | | 865m | 868m | | | 854w | 856w | |
| 840 | 840 | 838vw | 833vw | 831 | 831 | 844w | 845w | $\delta_s(NO)_\phi+\tau(\phi)$ |
| | | | | 823 | 823 | | | |
| 810 | 810 | | 810vw | 811 | 811 | 813vw | | $\delta(CH)_\phi$ |
| 807 | 807 | 807m | | | | | | $\nu(CNHC)$ |
| 794 | 794 | | | 798 | 798 | | | $\rho(CH_3)$ |
| 790 | 790 | | | | | | | $\nu(CNHC)_{\phi+\theta}+\delta_s(NO)_\phi$ |
| 775 | 775 | 777m | 761vw | | | | | $\rho(CH_3)$ |
| | | 757s | | 749 | 749 | 751s | 759vw | |
| 735 | 735 | | | 735 | 735 | | 745vw | $\delta(CH)_\theta$ |
| | | | | 728 | 728 | | | |
| 719 | 719 | 720w | 724w | 724 | 724 | | | $\delta_{as}(NO)_\phi$ |
| 707 | 707 | | | 710 | 710 | | | $\delta(NH)$ |
| 699 | 699 | 691m | | | | 694m | | $\delta(CNHC)_{\phi+\theta}$ |
| 672 | 672 | 682m | | 674 | 674 | 679w | 681m | $\delta(CH)_\theta$ |
| | | | | 665 | 665 | | | |
| 610 | 610 | 618vw | 620w | 614 | 614 | | 628w | $\tau(\phi_\theta)$ |
| 590 | 590 | 600w | 600w | 606 | 606 | | 592m | $\tau(\phi)+\tau(\theta)$ |
| 580 | 580 | 585w | | 578 | 578 | | | $\rho(CH_3)$ |
| 531 | 531 | 536w | 541m | 522 | 522 | 534w | 536vw | $\gamma(\phi)+\nu_{as}(NO)_\phi$ |
| 522 | 522 | 510m | 510w | | | 512s | 511vw | $\delta(\phi)$ |
| 492 | 492 | 470w | | 493 | 493 | 458w | | $\delta(CH)_\theta$ |
| 463 | 463 | | | 430 | 430 | 436w | 436vw | $\rho(CH_3)$ |
| 401 | 401 | | 403w | 401 | 401 | 419w | | $\delta(CH)_\theta$ |
| 395 | 395 | | 386w | 386 | 386 | 394w | 395vw | $\delta(CCH_3)+\delta(CNO)_\phi$ |
| 373 | 373 | | | 367 | 367 | 375w | 383w | $\delta(\phi)+\nu(C\text{-}NO_2)$ |
| | | | | | | 353w | | |
| | | | | | | 342w | | |
| | | | | | | 335w | | |
| | | | 331w | | | 328w | | |
| | | | | | | 324w | 323w | |
| | | | | 311 | 311 | 315vw | | |
| 308 | 308 | | 301w | | | 303m | | |
| 290 | 290 | | 289w | | | 295m | 295vw | $\delta(CNHC)_{\phi+\theta}$ |
| | | | | 280 | 280 | 282m | | |
| | | | | 250 | 250 | 278m | | $\tau(CH_3)$ |
| 266 | 266 | | | 248 | 248 | 266s | 264vw | |
| | | | 242w | | | 254w | | |
| | | | | | | 248w | | |
| 231 | 231 | | 210w | | | 245w | | |
| 224 | 224 | | | | | 227w | | |

| | | | | | | |
|---|---|---|---|---|---|---|
| | | | | 212m | 210w | |
| 205 | 205 | 205 | 205 | 208m | | |
| | | | | 203m | | |
| | | | | 194w | | |
| 187 | 187 | 184 | 184 | 188w | | $\delta(CNHC)_{\phi+\theta}$ |
| 107 | 107 | 94 | 94 | 177w | | $\delta(CNHC)_{\phi+\theta}$ |
| 83 | 83 | 84 | 84 | 150m | | $\delta_s(NO)_{\phi}+\delta(CNHC)_{\phi+\theta}$ |
| | | 79 | 79 | 122m | 107m | |
| 62 | 62 | 55 | 55 | | | $\nu(NO)_{\phi}$ |
| 38 | 38 | 42 | 42 | | 77m | |
| 19 | 19 | 20 | 20 | | 54s | |

**Figure S1**. The deconvolution of 2DFP plot for individual interactions in the crystal of PA3N4MP.

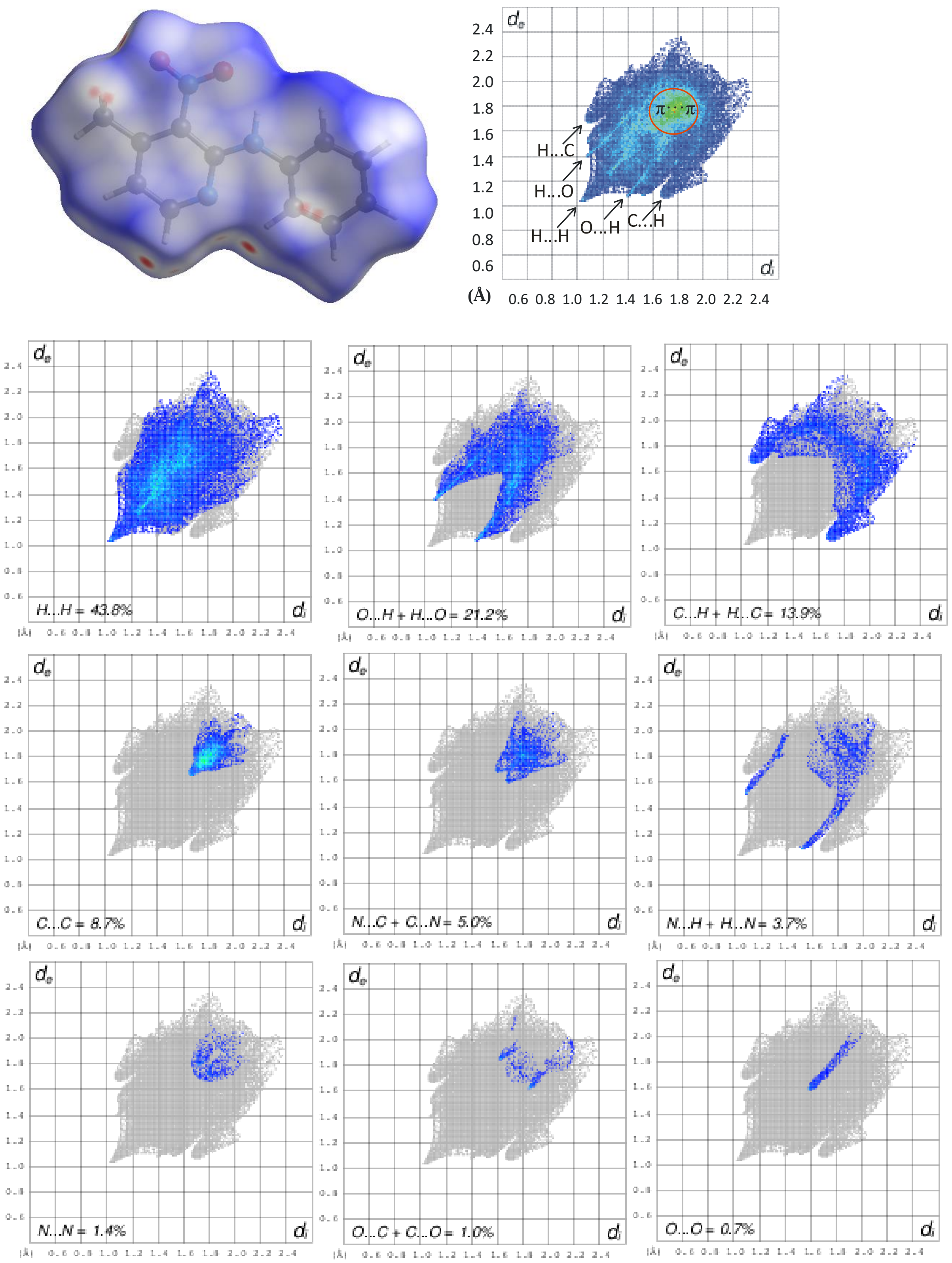

**Figure S2**. The deconvolution of 2DFP plot for individual interactions in the crystal of PA3N6MP.

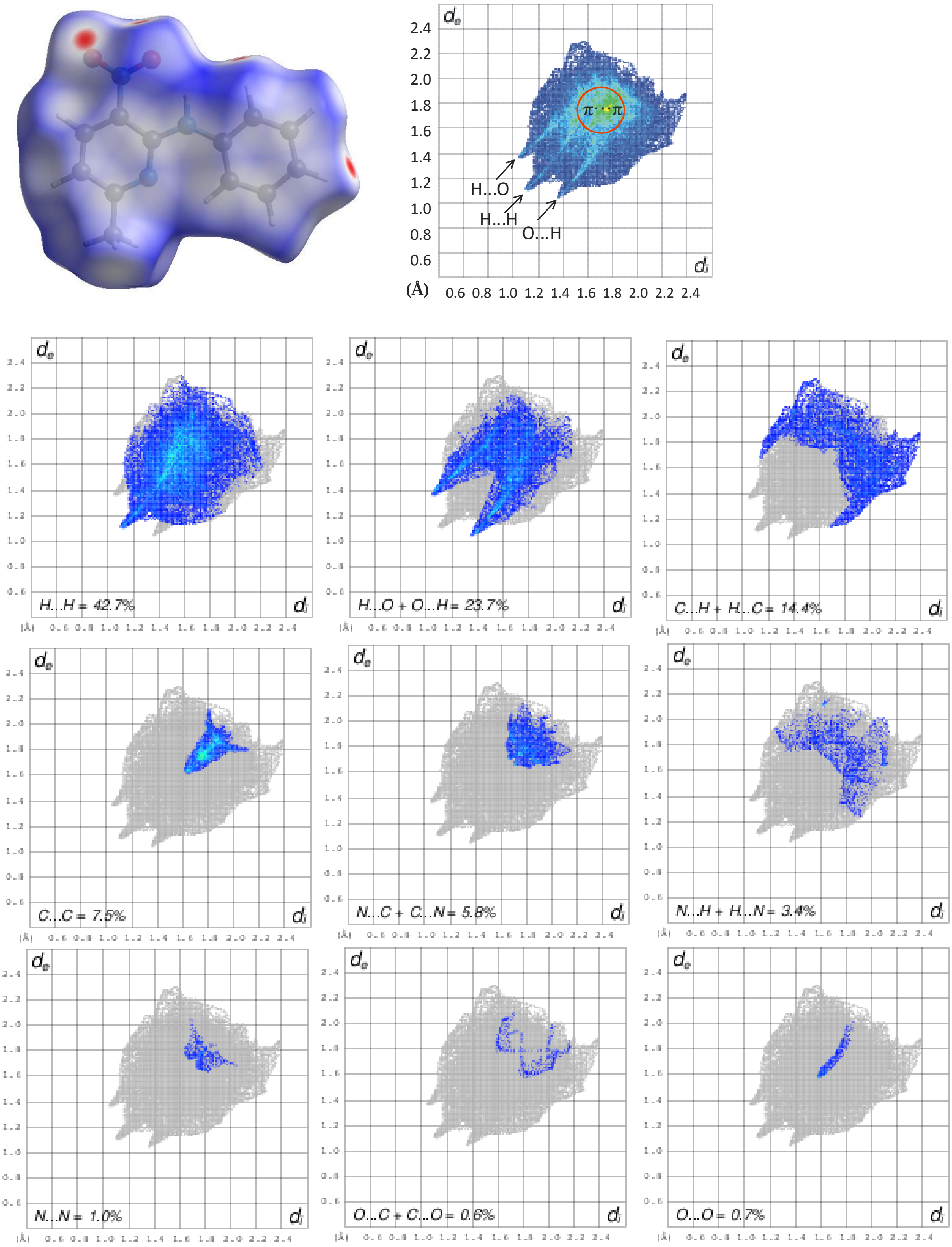

**Figure S3.** $^1$H NMR spectra of the studied isomers: A) 2-*N*-phenylamino-4-methyl-3-nitro- and B) 2-*N*-phenylamino-6-methyl-3-nitro- pyridines.

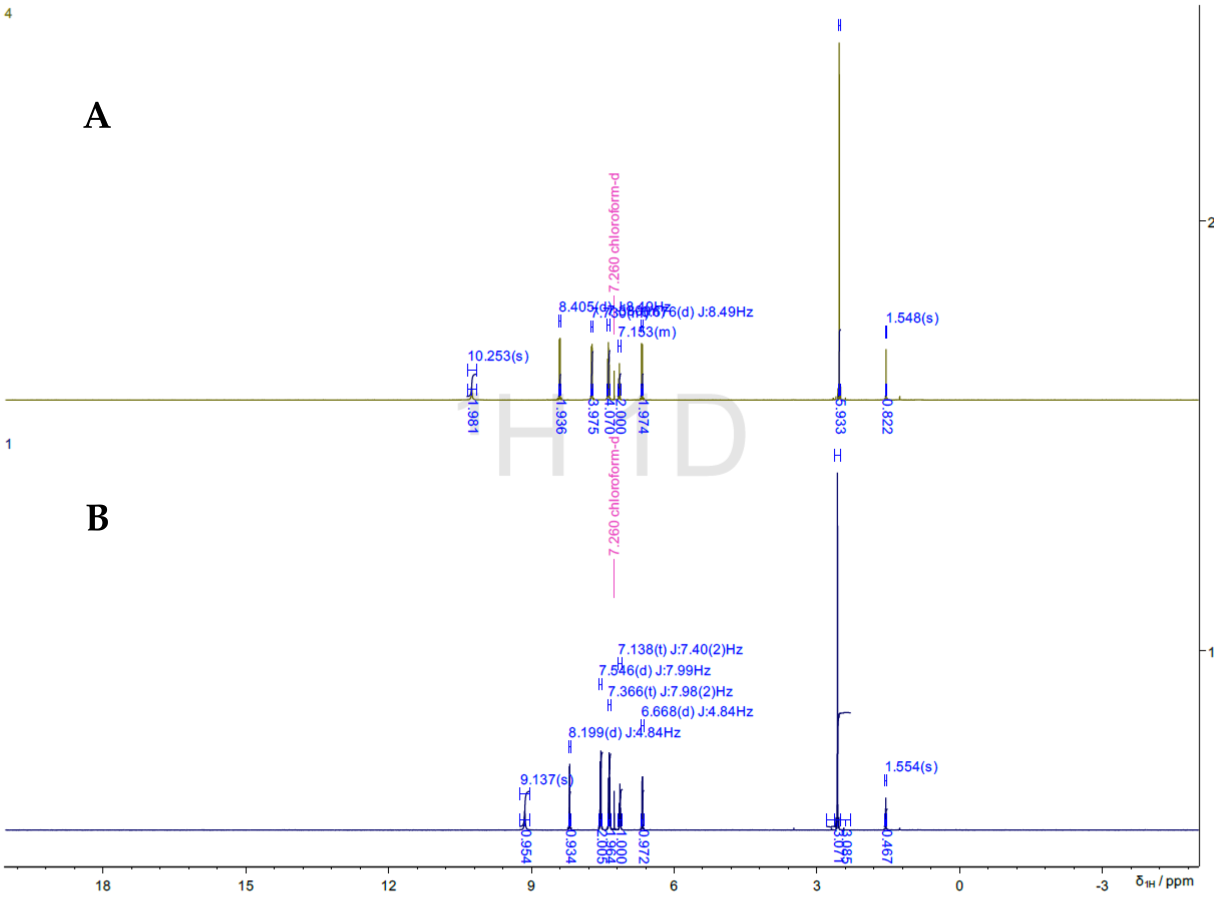

**Figure S4**. $^{13}$C NMR spectra of the studied isomers: A) 2-*N*-phenylamino-4-methyl-3-nitro- and B) 2-*N*-pheny lamino-6-methyl-3-nitro- pyridines.

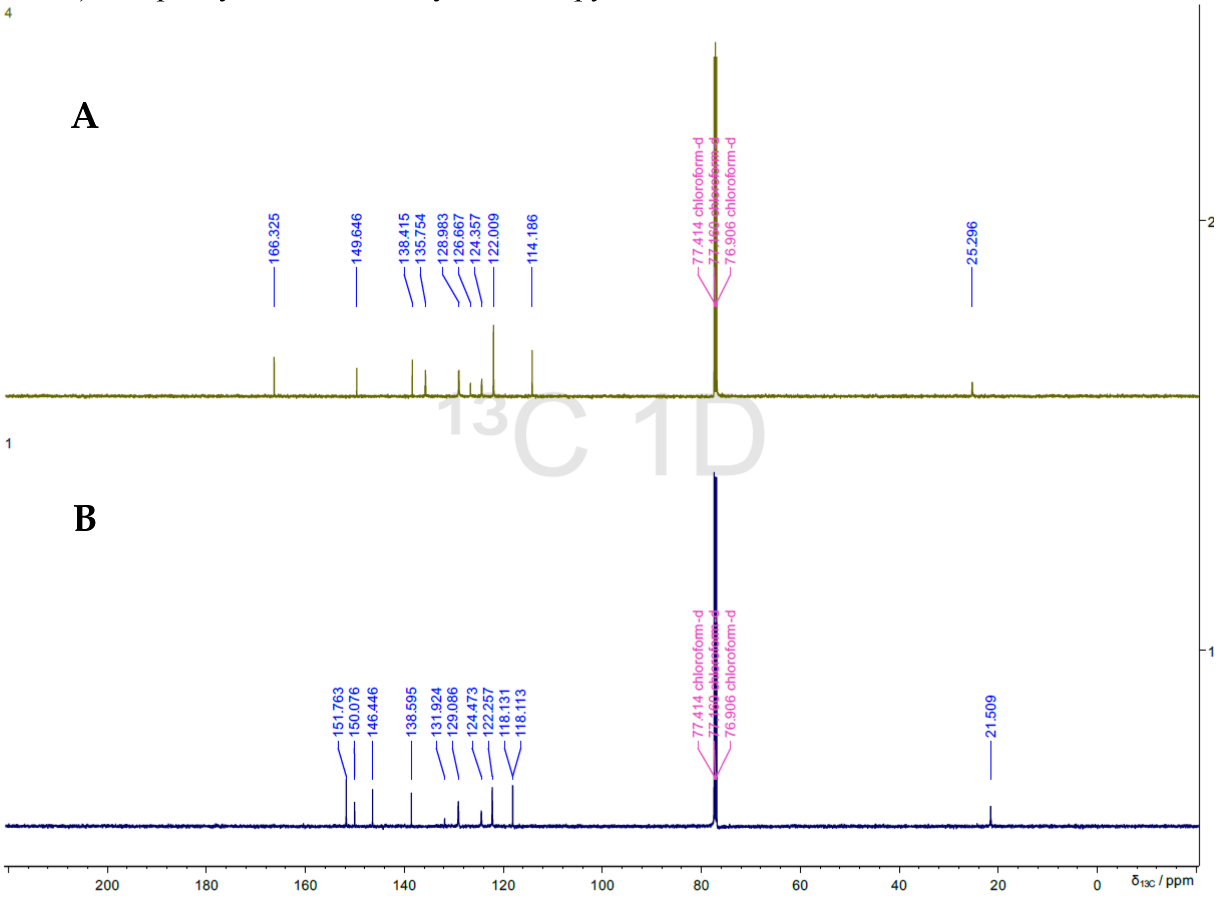

Supplement: Supplementary file 1 [file ijms-26-02874-s001.zip › ijms-3493985-supplementary.pdf]
